# Heritable maintenance of chromatin modifications confers transcriptional memory of interferon-γ signaling

**DOI:** 10.1038/s41594-025-01522-8

**Published:** 2025-04-04

**Authors:** Pawel Mikulski, Sahar S. H. Tehrani, Anna Kogan, Izma Abdul-Zani, Emer Shell, Louise James, Brent J. Ryan, Lars E. T. Jansen

**Affiliations:** 1 Department of Biochemistry, University of Oxford, Oxford, UK; 2 The International Institute of Molecular Mechanisms and Machines PAS, Warsaw, Poland; 3 Instituto Gulbenkian de Ciência, Oeiras, Portugal; 4 Department of Physiology, Anatomy & Genetics, University of Oxford, Oxford, UK

**Keywords:** Gene silencing, Epigenomics, Chromatin structure, Histone post-translational modifications

## Abstract

Interferon-γ (IFNγ) transiently activates genes related to inflammation and innate immunity. A subset of targets retain a mitotically heritable memory of prior IFNγ exposure, resulting in hyperactivation upon re-exposure through poorly understood mechanisms. Here, we discover that the transcriptionally permissive chromatin marks H3K4me1, H3K14ac and H4K16ac are established during IFNγ priming and are selectively maintained on a cluster of guanylate-binding protein (GBP) genes in dividing human cells in the absence of transcription. The histone acetyltransferase KAT7 is required for H3K14ac deposition at GBP genes and for accelerated GBP reactivation upon re-exposure to IFNγ. In naive cells, the GBP cluster is maintained in a low-level repressive chromatin state, marked by H3K27me3, limiting priming through a PRC2-dependent mechanism. Unexpectedly, IFNγ priming results in transient accumulation of this repressive mark despite active gene expression. However, during the memory phase, H3K27 methylation is selectively depleted from primed GBP genes, facilitating hyperactivation. Furthermore, we identified a *cis*-regulatory element that forms transient, long-range contacts across the GBP cluster and acts as a repressor, curbing hyperactivation of previously IFNγ-primed cells. Our results provide insight into the chromatin basis for the long-term transcriptional memory of IFNγ signaling, which might contribute to enhanced innate immunity.

## Abstract (web summary)

Mikulski et al. identify a distinct chromatin structure at clustered GBP innate immune genes that preserves a mitotically heritable memory of prior priming by interferon-γ.

## Main

Cells can respond to a multitude of stimuli by rewiring their gene expression programmes. Although acute activation of transcription in response to external signals is well understood, the longer-term cellular consequences of such signals remain less clear. Cells can retain a memory of past stimulation that can be passed down through several generations. This post-stimulus epigenetic memory has been characterized primarily in the context of long-term gene repression, involving read–write mechanisms that maintain DNA methylation, repressive histone modifications and Polycomb complex binding^1^. However, transient gene activation can also be remembered, resulting in what is known as long-term transcriptional memory^2^. This memory of gene activation is relevant because it can alter the cellular response to future re-exposure to activating signals.

Transcriptional-memory phenomena have been observed across multiple cellular processes and species^3^, yet their molecular mechanisms remain poorly understood. Cellular exposure to the cytokine IFNγ is known to induce transcriptional memory, making it an ideal model for exploring underlying mechanisms. IFNγ activates a broad set of genes related to inflammation, cell death and host defense against pathogens and cancer^4^. In addition to transiently activating many genes, IFNγ also induces long-term transcriptional memory for a subset of genes in different cell types, including innate immune macrophages, non-immune fibroblasts and cancer cells^5–8^. We and others have demonstrated that genes involved in memory tend to cluster together in the genome^5,8^. One such cluster is a family of genes encoding GBPs, GTPases that are strongly activated by type II IFNs^8^ and more transiently by type I IFNs^9,10^. GBPs are crucial for inflammasome activation and have a key role in protecting against infections and cancer^11^. Although IFNγ strongly activates GBP genes, expression is lost by 2 days following an IFNγ pulse; however, cells maintain a heritable epigenetic memory of activation for up to 14 days of continued proliferation without target gene expression^8^. This primed state results in hyperactivation of GBP genes upon re-exposure to IFNγ, which could represent a crucial means for enhanced innate immune responses to repeated cellular insults.

Previous studies in yeast, plants and mice have highlighted the involvement of *trans*-acting transcription factors that persist after priming^12–14^. For instance, in mice, temporary skin inflammation results in long-term retention of AP1 transcription factors at target promoters, sensitizing cells for future challenge^14^. We previously examined *trans*-acting factors in the context of IFNγ-mediated transcriptional memory^8,15^ and found that although GBP expression depends on the transcription factors STAT1 and IRF1, their expression is not retained in cellular memory^8^. STAT1, although essential for priming, is not necessary for the primed state to be maintained^15^. These earlier results suggest that a *cis*-acting chromatin-based mechanism could contribute to memory, even in the absence of ongoing binding of transcription factors.

## Results

### Permissive chromatin modifications are heritably maintained at GBP genes

**Fig. 1:**
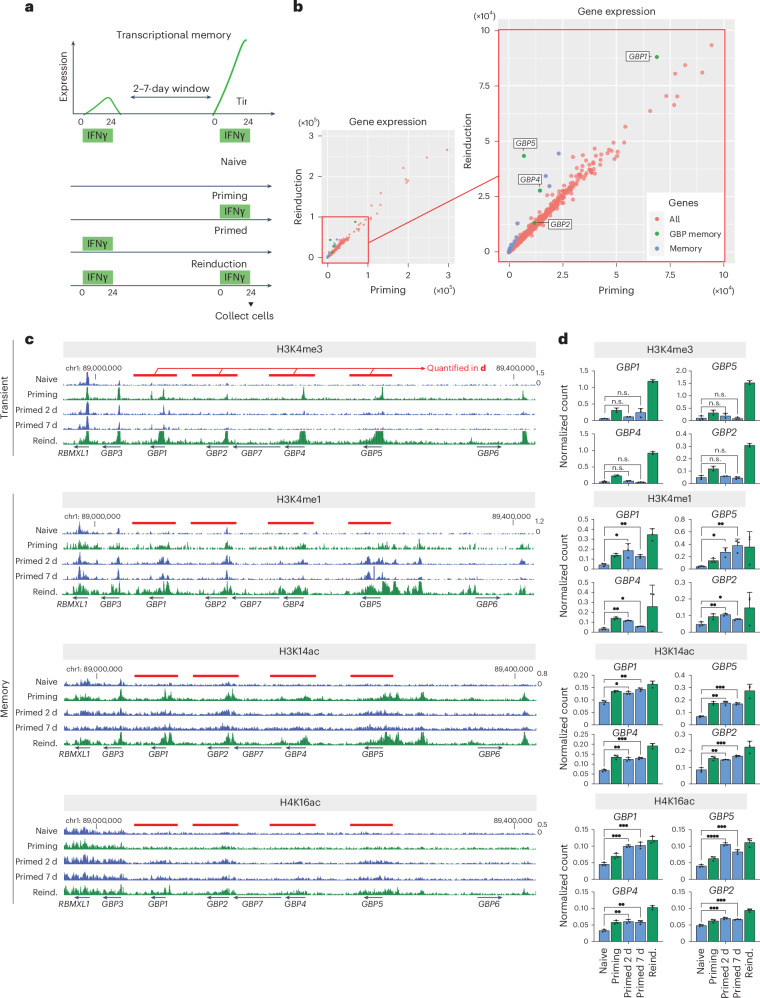
Specific permissive chromatin modifications are established during priming and are heritably maintained at GBP memory genes post-stimulation. **a**, The experimental transcriptional memory regime outlining the timing of IFNγ incubation and cell collection. **b**, Gene expression plots for IFNγ-mediated stimulation comparing priming versus reinduction. The plot on the right shows a more detailed view of the boxed area in the left panel. Each dot corresponds to an individual IFNγ-stimulated gene, color-coded according to the legend. Data are reanalyzed from ref. ^8^. **c**, Cut&Run-seq enrichment of permissive chromatin modifications in the IFNγ-induced transcriptional memory regime, represented as genome browser snapshots over the GBP cluster. Red bars indicate regions over GBP memory genes that were used for quantification. Reind., reinduced. **d**, Quantification of normalized Cut&Run sequencing reads for respective chromatin modifications over GBP memory genes. The error bars correspond to s.e.m. (*n* = 3). *n* values for all panels correspond to biological replicates. n.s. > 0.05; •, *P* ≤ 0.05; ••, *P* ≤0.01; •••, *P* ≤ 0.001; ••••, P ≤0.0001. Statistical significance was calculated using the two-sided *t*-test, and prior determination of homo- or heteroscedasticity was done with the with *F*-test. Source data

**Extended Data Fig. 1:**
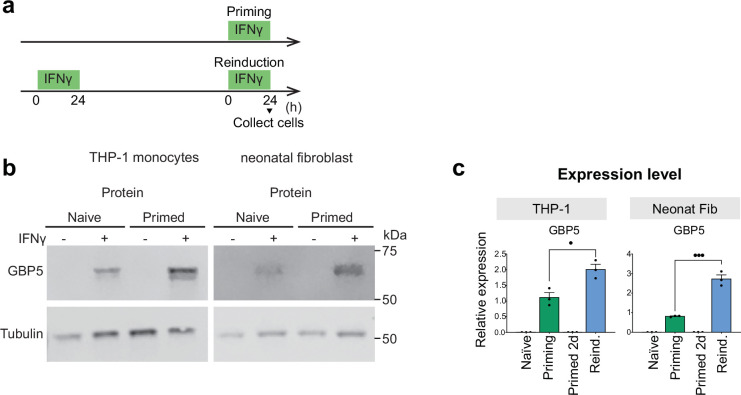
Human primary fibroblasts and THP-1 cells display transcriptional memory of GBP genes. **a**, Experimental regime outlining timing of IFNγ-incubation and cell harvesting**. b**, Indicated cells were subjected to IFNγ-regime as in a and cell extracts prepared for SDS-PAGE and immunoblotting. Blots were incubated with GBP5 antibody to assess protein levels. α-Tubulin (Tubulin) was used as a loading control. **c**, Expression of GBP1 and 5 in indicated cells during IFNγ priming or reinduction measured by RT-qPCR. Error bars correspond to SEM (*n* = 3). *n* value corresponds to biological replicates (separate cell populations in tissue culture plates). *P* values for relevant pairwise comparisons are indicated: n.s. > 0.05, • ≤0.05, •• ≤0.01, ••• ≤0.001, •••• ≤0.0001. Statistical significance was calculated with two-sided *t*-test and prior determination of homo- or heteroscedasticity with F-test. Source data

To discover potential carriers of mitotically heritable transcriptional memory of IFNγ gene activation, we exposed human HeLa cells to recurrent IFNγ stimulation (priming and reinduction), with intervals of days or up to a week without a stimulus while allowing for continuous cell proliferation (Fig. 1a). Analysis of our previously reported RNA-sequencing dataset^8^ revealed that the most IFNγ-inducible genes are activated to similar levels regardless of prior activation, indicating no memory of previous exposure. However, a subset of genes shows hyperactivation of expression upon reinduction compared with priming (Fig. 1a,b), as has been previously described^5,8^. The most prominent among these is the GBP family of genes, in which *GBP1*, *GBP5* and *GBP4* (the strong memory genes) exhibit the highest degree of hyperactivation, whereas *GBP2* shows weak hyperactivation (the weak memory gene). Notably, all these GBP genes are paralogs that cluster together on human chromosome 1. These findings suggest that an initial IFNγ stimulation (priming) establishes a state in this cluster that enables faster and stronger expression of GBP memory genes upon IFNγ re-exposure (reinduction) (Fig. 1a,b). Between IFNγ pulses, cells continuously proliferate without expression of GBP genes, suggesting an epigenetic mode of transcriptional memory. We also observed IFNγ-induced transcriptional memory in the THP-1 human monocyte cell line and in human primary foreskin fibroblasts. Both *GBP1* and *GBP5* exhibited a similar pattern of inducibility and hyperactivation in primed cells (Extended Data Fig. 1), indicative of transcriptional memory in both non-transformed and innate immune cell types. This finding is consistent with observations in mouse fibroblasts and macrophages^5^.

Given the heritable nature of gene priming, we explored changes in specific *cis*-acting chromatin modifications, focusing on their potential retention through cell division and their association with active genes. We previously found that increased chromatin accessibility, H3K27 acetylation, H3K4 dimethylation and H3K36 trimethylation are transiently associated with GBP genes but return to baseline levels upon loss of expression^8^.

**Extended Data Fig. 2:**
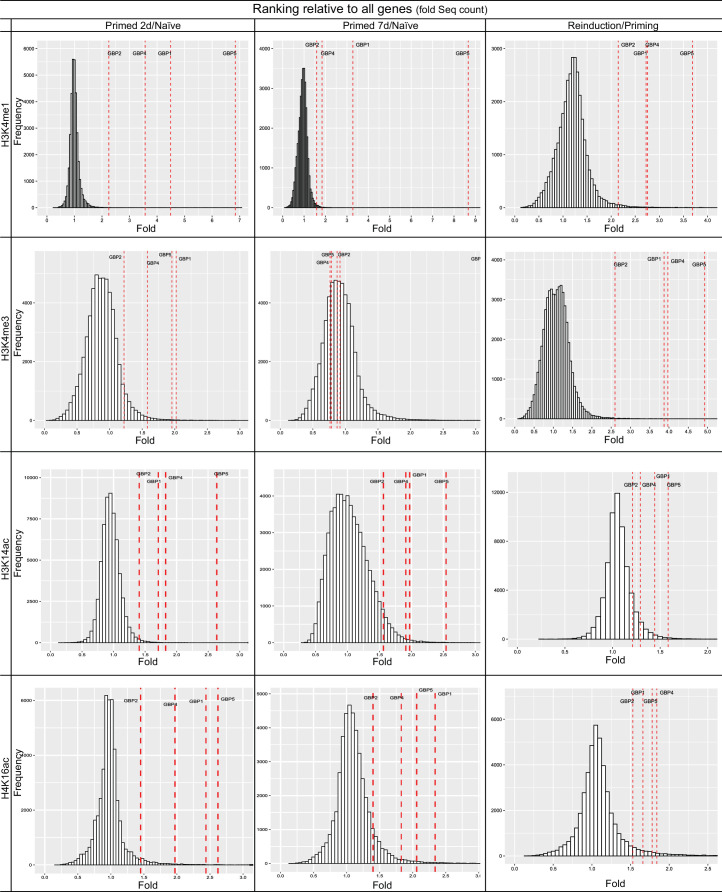
GBP memory genes exhibit selective retention of specific permissive chromatin modifications in IFNγ transcriptional memory. Histograms for fold changes between indicated conditions for selected permissive chromatin modifications. The data corresponds to Cut&Run sequencing read count calculated genome-wide for each human gene (hg38 reference) with the enrichment over respective gene body and regions: 3 kb upstream of transcriptional start site (promoter region) and 3 kb downstream of transcriptional termination site (terminator region). Dashed vertical lines mark folds corresponding to GBP memory genes.

Here, we explored the roles of histone H3K4 mono- and trimethylation and H3K14 and H4K16 acetylation because these modifications are known to regulate active chromatin states^16–19^. We assessed their enrichment in chromatin using Cut&Run-seq^20^ in naive cells, during IFNγ stimulation, in the post-stimulation (primed) period and upon reinduction. Naive cells exhibit basal levels of these modifications, which accumulate at GBP promoters and bodies upon IFNγ induction and further increase upon reinduction (Fig. 1c,d), correlating with gene expression (Fig. 1a). The enhanced accumulation of these modifications at GBP genes, relative to the level observed during priming, is among the highest observed in the genome (Extended Data Fig. 2). Once IFNγ is removed, levels of H3 trimethylated at K4 (H3K4me3) rapidly decline in primed cells. Little remains after 2 days, and prestimulation levels are reached by 7 days post-IFNγ washout (Fig. 1c,d), similar to our earlier report on H3 dimethylated at K4 (H3K4me2)^8^. This indicates that both H3K4me2 and H3K4me3 are acute, non-memorized modifications associated with ongoing expression that is reset when transcription ceases.

In striking contrast, we observed that H3 monomethylated at K4 (H3K4me1), H3 acetylated at K14 (H3K14ac) and H4 acetylated at K16 (H4K16ac) were all maintained at higher levels in primed cells than in naive cells. The retention was most pronounced on the strong memory genes compared with the weak memory gene (Fig. 1c,d) and the rest of the genome (Extended Data Fig. 2). This finding suggests that H3K4me1, H3K14ac and H4K16ac are selectively retained on the chromatin of memory GBPs in cells previously exposed to IFNγ, despite the lack of GBP expression and continuous cell proliferation for up to 7 days (or ~7 cell divisions). Our results indicate that retaining unique permissive chromatin modifications post-stimulation could confer transcriptional memory, enabling differential expression of memory genes in response to recurrent stimulations.

### IFNγ drives transient repressive chromatin assembly at GBP cluster

**Fig. 2:**
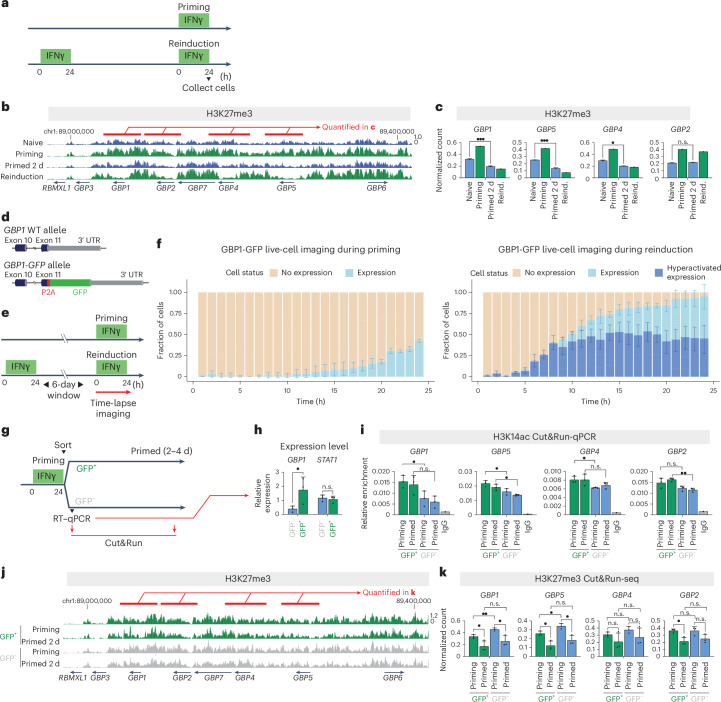
The IFNγ-activated GBP cluster accumulates repressive chromatin that is selectively removed from GBP memory genes post-stimulation. **a**, The experimental transcriptional memory regime, outlining the timing of IFNγ incubation and cell collection**. b**, Cut&Run-seq enrichment of H3K27me3 over the GBP cluster, during the IFNγ-induced transcriptional memory regime. The red bars indicate the regions over GBP memory genes that were used for quantification. **c**, Quantification of normalized H3K27me3 Cut&Run sequencing reads over GBP memory genes (*n* = 3 (naive, primed); *n* = 2 (priming, reinduction)). **d**, A schematic of the endogenous *GBP1* gene structure in the *GBP1-GFP* reporter line. WT, wild type. **e**, Experimental regime outlining timing of IFNγ-incubation and time-lapse imaging **f**, Time-lapse of live-cell GBP1–GFP protein expression during priming (left) and reinduction (6 days after priming) (right). The fraction of cells displaying expression or hyperactivated expression of GBP1–GFP is plotted for each time point. Hyperactivated expression during reinduction is defined as levels above those observed during priming (Methods). The bars represent the mean and s.d. of three biological replicates. **g**, The experimental regime outlining the timing of IFNγ incubation, sorting of GFP^+^ and GFP^–^ cells and downstream analysis. **h**, Normalized expression of the indicated target genes following IFNγ priming and sorting of GFP^+^ and GFP^–^ cells, measured by RT–qPCR (*n* = 3). **i**, Quantification of H3K14ac Cut&Run–qPCR over the indicated loci following IFNγ priming and sorting of GFP^+^ and GFP^–^ cells and samples immediately following sorting (priming) and 4 days following sorting (primed) (*n* = 3, except IgG (*n* = 1)). **j**, Cut&Run-seq enrichment of H3K27me3 over the GBP cluster in IFNγ-induced cells sorted for GFP^+^ and GFP^–^ expression. Red bars indicate the regions over GBP memory genes that were used for quantification. **k**, Quantification of normalized Cut&Run sequencing reads for respective chromatin modifications over GBP memory genes (*n* = 3). The error bars in **c**, **h**, **i** and **k** represent the s.e.m. (*n* = 3). *n* values for all panels correspond to biological replicates. n.s. > 0.05; •, *P* ≤ 0.05; ••, *P* ≤0.01; •••, *P* ≤ 0.001; ••••, P ≤0.0001. Statistical significance was calculated using the two-sided *t*-test, and prior determination of homo- or heteroscedasticity was done with the *F*-test. Source data

**Extended Data Fig. 3:**
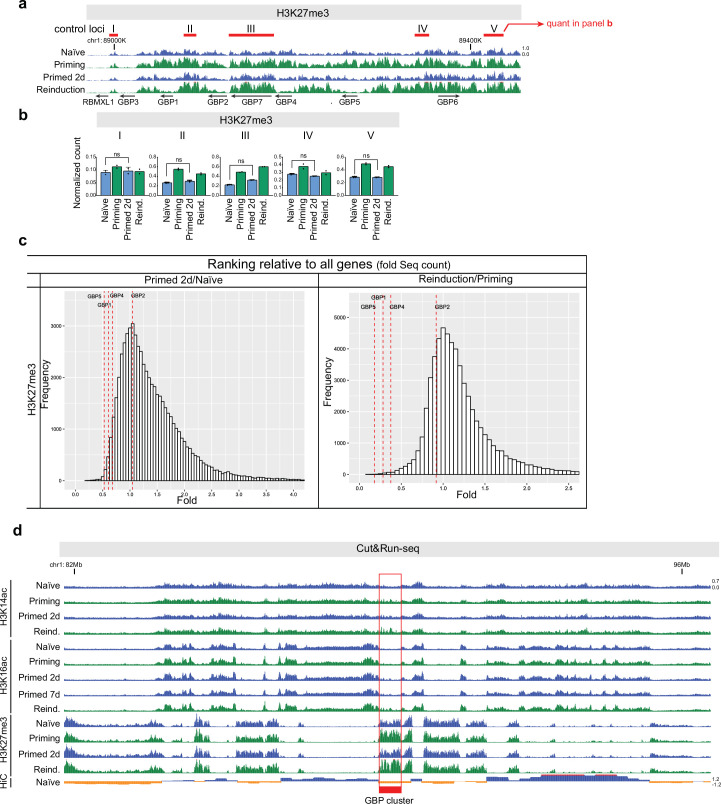
Repressive H3K27me3 chromatin is broadly accumulated across the GBP cluster but selectively removed from GBP genes in response to IFNγ. **GBP cluster-wide analysis. a**, Cut&Run-seq enrichment of H3K27me3 in IFNγ-induced transcriptional memory regime represented as genome browser snapshots over GBP cluster. Red bars indicate regions outside of GBP memory genes broadly distributed in the cluster used for read quantification. **b**, Quantification of normalized Cut&Run sequencing reads for respective chromatin modifications over GBP cluster outside of GBP memory genes. Error bars correspond to SEM (*n* = 3). Sample size was *n* = 3 (Naïve and Primed 2 d) or *n* = 2 values (Priming and Reinduction). *n* value corresponds to biological replicates. Error bars correspond to SEM (*n* = 2 or 3). *P* values for relevant pairwise comparisons are indicated: n.s. > 0.05, • ≤0.05, •• ≤0.01, ••• ≤0.001, •••• ≤0.0001. Statistical significance was calculated with two-sided *t*-test and prior determination of homo- or heteroscedasticity with F-test. **c, Gene-specific, genome-wide analysis**. Histograms of fold changes between indicated conditions for H3K27me3. The data corresponds to Cut&Run sequencing read count calculated genome-wide for each human gene (hg38 reference) with the enrichment over respective gene body and regions: 3 kb upstream of transcriptional start site (promoter region) and 3 kb downstream of transcriptional termination site (terminator region). Dashed vertical lines mark folds corresponding to GBP memory genes. **d, GBP cluster resides in a repressive topological chromatin domain**. Multi-megabase visualization of Cut&Run-seq enrichment of selected permissive/repressive broad-distribution chromatin modifications and A/B compartmentalization (labeled blue and orange, respectively) from HiC assays^22^. Red frame indicates the position of the GBP cluster. Source data

In addition to the propagation of permissive chromatin marks, the removal of repressive marks might also have a role in maintaining a primed state. To investigate this, we analyzed the Polycomb-mediated repressive histone modification H3 trimethylated at K27 (H3K27me3)^21^ using Cut&Run-seq. We found that it was broadly associated with the GBP cluster in naive cells, including intergenic regions, in agreement with the lack of GBP expression before stimulation (Fig. 2a–c). Surprisingly, although H3K27me3 is known to repress transcription^21^, we found that it accumulates across the cluster during priming (Fig. 2a–c), coinciding with upregulation of GBP genes (Extended Data Fig. 3a,b). After priming, H3K27me3 is locally depleted from gene bodies and proximal promoters of strong memory genes (Fig. 2a–c). This loss is maintained during memory and becomes even more pronounced during reinduction. Genome-wide analysis revealed that the selective loss of H3K27me3 under primed and reinduction conditions is most prominent at GBP memory genes (Extended Data Fig. 3c). The limits of this H3K27me3 repressive domain coincide with previously reported topologically associating domain (TAD) borders around the GBP cluster^22,23^, overlapping with a B compartment as defined by Hi-C analysis^22^. This domain shows overall higher levels of H3K27me3 than the cluster-wide permissive modifications H3K14ac and H4K16ac (Extended Data Fig. 3d), suggesting that the GBP cluster resides in a generally repressive chromatin domain.

**Extended Data Fig. 4:**
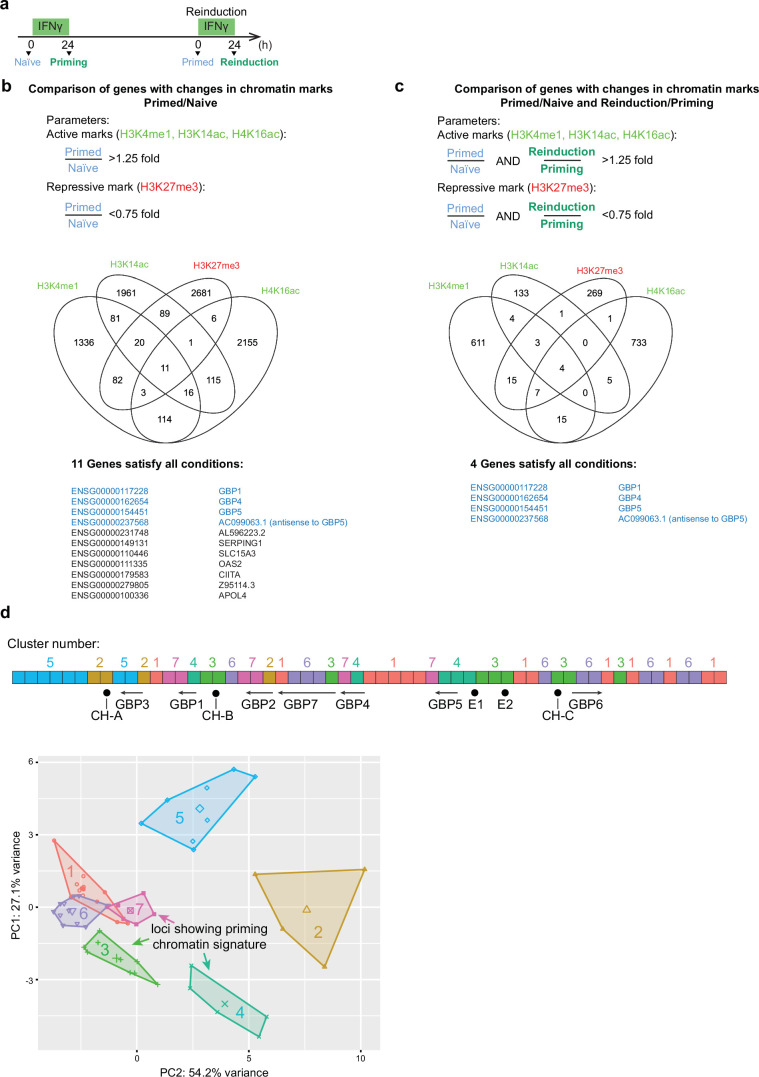
Selected genes display unique chromatin signatures during priming, the primed state and reinduction. **a**, Experimental transcriptional memory regime outlining timing of IFNγ incubation and cell harvesting. **b**, Venn diagram representing overlaps between genes with the same directional changes of selected memory chromatin marks in comparison: primed/naïve. The list below the diagram represents Ensembl ID and names of the genes from shared overlap between all conditions. **c**, Venn diagram representing overlaps between genes with directional changes of selected memory chromatin marks in comparison: primed/naïve and reinduction/priming. The list below the diagram represents Ensembl ID and names of the genes from shared overlap between all conditions. Gene loci within the GBP cluster are indicated in blue. **d**, K-means clustering analysis of chromatin signature within the GBP cluster. 10 kb bins across the 570 kb GBP TAD and its neighbouring regions were analysed for common patterns of enrichment of heritable chromatin marks (H3K4me1, H3K14ac, H4K16ac and H3K27me3) across conditions: Naïve, Primed 2 d, Priming, Reinduction. Cluster number was pre-determined with 3 independent methods. The bins group into 7 discernible clusters. Top: annotation and mapping of cluster identities onto genomic coordinates within the GBP cluster, color-coded as in PCA plot (CH-A, B, C are Cohesin binding sites described in^8^. E1 and E2 are cis-regulatory elements that bind STAT1^15^). Bottom: PCA analysis visualizing clustering of the bins. Source data

We analyzed the genome for the frequency of this specific pattern of chromatin modifications and found that only 11 genes exhibited a simultaneous increase in permissive chromatin modifications (H3K4me1, H3K14ac and H4K16ac) during reinduction compared with priming while also losing H3K27me3. When this analysis was combined with naive and primed conditions, only four genes showed the same pattern (Extended Data Fig. 4), all of which are located in the GBP cluster.

**Extended Data Fig. 5:**
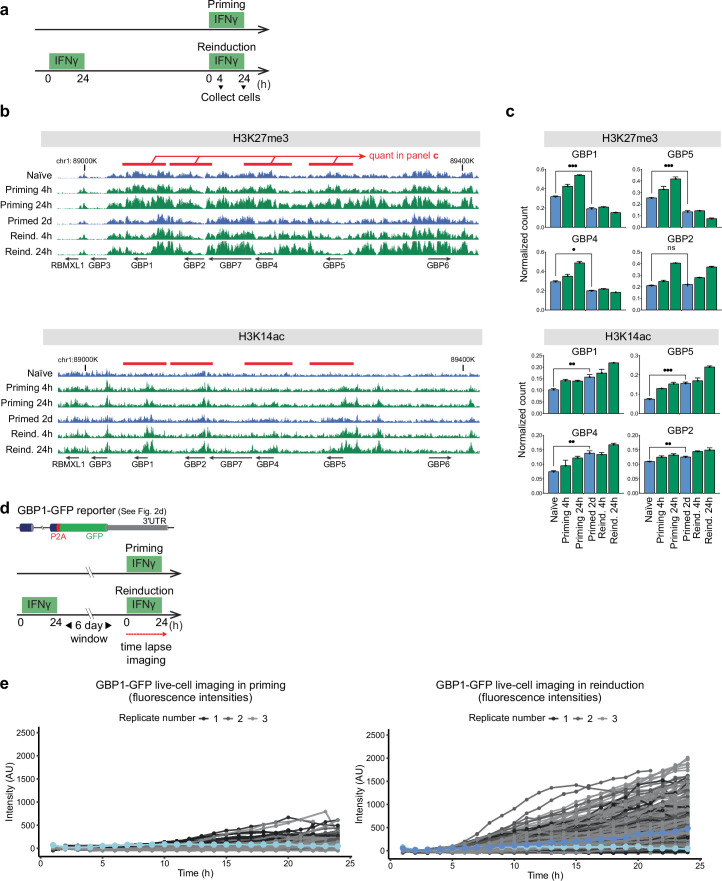
Temporal dynamics of H3K14ac and H3K27me3 accumulation. **a**, Experimental transcriptional memory regime outlining timing of IFNγ-incubation and cell harvesting**. b**, Cut&Run-seq enrichment of indicated chromatin modifications during transcriptional memory regime with short (4 h) and long (24 h) IFNγ stimulation. The enrichment is represented as genome browser snapshots over GBP cluster. Red bars indicate regions over GBP memory genes used for quantification. **c**, Quantification of normalized Cut&Run sequencing reads for respective chromatin modifications over GBP memory genes with short (4 h) and long (24 h) IFNγ stimulation. Error bars correspond to SEM (*n* = 3). *n* value corresponds to biological replicates. *P* values for relevant pairwise comparisons are indicated: n.s. > 0.05, • ≤0.05, •• ≤0.01, ••• ≤0.001, •••• ≤0.0001. Statistical significance was calculated with two-sided *t*-test and prior determination of homo- or heteroscedasticity with F-test. **d**, Schematic outline of GBP1-GFP reporter line and experimental scheme for IFNγ priming and reinduction followed by time lapse imaging. **e**, Time-lapse of live-cell GBP1-GFP protein expression during priming (left) and reinduction (6 days after priming) (right). Tracks of GFP intensities in individual cells were recorded over 24 h of IFNγ exposure. Shades of gray represent cells from individual experiments. Light blue lines represent the threshold of GFP intensity set to quantify the fraction of cells with GBP1-GFP expression above baseline (naïve) levels. Dark blue line represents the threshold of GFP intensity set to quantify the fraction of cells with hyperactivated GBP1-GFP expression above the level observed in cells during priming. Source data

To understand the relationship between repressive and permissive chromatin features at the GBP cluster, we directly compared H3K27me3 and H3K14ac, because the latter is efficiently maintained at IFNγ-primed cells (Fig. 1c,d). Analysis was conducted 4 h post-IFNγ stimulation and showed that both H3K14ac and H3K27me3 had already accumulated above naive levels, indicating that chromatin reorganization is rapid (Extended Data Fig. 5a–c). Of note, during priming, both H3K27me3 and H3K14ac accumulate on memory GBP genes. However, in primed cells, their local occupancy becomes antagonistic; the highest H3K14ac peaks overlap with regions of local H3K27me3 depletion (Extended Data Fig. 5b). This inverse enrichment persists during reinduction. These results suggest that, although permissive and repressive chromatin modifications at the GBP cluster are initially coenriched, they occupy locally distinct chromatin regions during the memory phase. The sustained presence of permissive modifications, coupled with the selective removal of repressive ones, could underpin the transcriptional memory of GBP genes.

### Repressive chromatin assembles at actively transcribing GBP genes

We have previously reported that priming leads to the activation of IFNγ-stimulated genes in all cells, indicating that clonal populations of cells are uniformly responsive to IFNγ^8^. However, despite all cells responding to IFNγ, we showed that GBP genes are activated in only a subset of IFNγ-primed cells, suggesting that transcriptional memory of GBP genes is partly due to an increased number of cells participating in expression following the initial priming event^8^. Therefore, the simultaneous increase of both transcriptionally permissive and repressive chromatin marks on GBP genes might result from a subpopulation of cells activating these genes and accumulating permissive chromatin while others silence the cluster and do not express GBP genes.

To test our hypothesis, we built a reporter cell line for IFNγ expression and memory that incorporates fluorescent protein (GFP) as part of the endogenous *GBP1* mRNA, expressed as a separate polypeptide to accurately reflect GBP1 expression in HeLa cells (Fig. 2d). This single-cell reporter enabled us to determine through live-cell imaging whether IFNγ priming and subsequent reinduction results in GBP1 expression in all or only a fraction of cells (Fig. 2e). We found that GBP1–GFP is modestly activated during priming but hyperactivated during reinduction of previously primed cells, even after a week of proliferation (Extended Data Fig. 5d,e). Notably, only ~30% of cells express detectable levels of GBP1 during the initial priming. This figure rises to more than 90% during reinduction (Fig. 2f).

**Extended Data Fig. 6:**
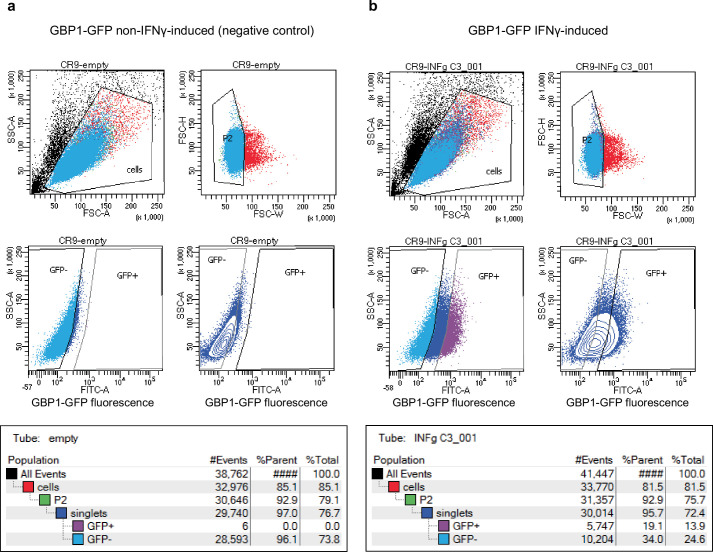
FACS gating strategy for experiments in Fig. 2i. Representative gating reports for GBP1-GFP samples - non-IFNγ-induced (negative control) (**a**) or IFNγ-induced (**b**). ‘Cells’ - initially selected cell population; P2 – singlet cell population; ‘GFP + ‘ – GFP-positive cell population; ‘GFP-’ – GFP-negative cell population.

**Extended Data Fig. 7:**
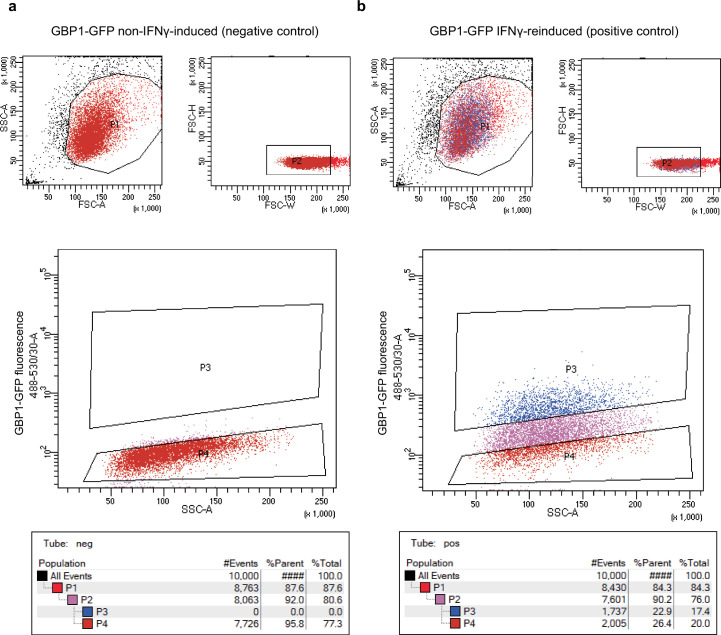
FACS gating strategy for experiments in Fig. 2j,k and Fig. 3i. Representative gating reports for GBP1-GFP samples - non-IFNγ-induced (negative control) (**a**) or IFNγ-reinduced (positive control) (**b**). P1 - initially selected cell population; P2 – singlet cell population; P3 – GFP-positive cell population; P4 – GFP-negative cell population.

To determine whether the accumulation of permissive and repressive marks stems from differential GBP expression across the cell population, we sorted GFP-positive and GFP-negative cells immediately after initial priming (Fig. 2g). Reverse transcription real-time quantitative PCR (RT–qPCR) confirmed that both GFP^–^ and GFP^+^ cells induce STAT1, indicating that both populations were responsive to IFNγ. As expected, *GBP1* mRNA levels were low in GFP^–^ cells and four- to fivefold higher in GFP^+^ cells (Fig. 2h). We analyzed H3K14ac (with Cut&Run–qPCR) and H3K27me3 (with Cut&Run-seq) in GFP^+^ and GFP^–^ cells, both immediately after priming and in primed cells, 4 or 2 days following IFNγ washout, respectively. During priming, levels of H3K14 acetylation at GBP genes were higher in GFP^+^ cells than in GFP^–^ cells, correlating with GBP1 expression levels. Notably, the levels of H3K14 acetylation in both GFP^+^ and GFP^–^ cells were largely retained during the memory phase, reflecting a lasting change in chromatin status even in the low-expressing GFP^–^ cells (Fig. 2i and Extended Data Fig. 6). Of note, we also found that accumulation of H3K27me3 and subsequent removal in primed cells occurred in both GFP^+^ and GFP^–^ cells (Fig. 2j,k and Extended Data Fig. 7). These results demonstrate that the accumulation of repressive H3K27me3 at the GBP cluster is not due to silencing in selective cells, but rather coincides with active gene transcription.

### H3K14ac and H3K27me3 writers are required for GBP expression and memory

**Fig. 3:**
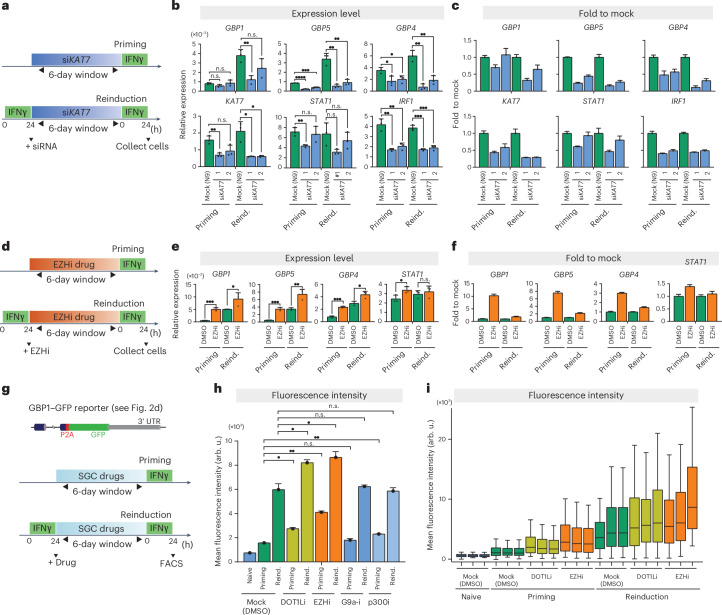
The writers for H3K14ac, H3K27me3 and H3K79me are functionally required for GBP gene expression and memory. **a**, The experimental scheme for transient KAT7 depletion during IFNγ priming and reinduction. **b**, Normalized RT–qPCR data for target genes upon IFNγ priming or reinduction after KAT7 depletion using two independent *KAT7*-targeted siRNAs compared with mock siRNA control (N9). **c**, Mean fold changes between si*KAT7*-1 or si*KAT7*-2 and mock control, derived from RT–qPCR data in **b**. **d**, The experimental scheme for EZH1/2 inhibition during IFNγ priming or reinduction. **e**, Normalized RT–qPCR data for target genes upon IFNγ priming or reinduction after EZH1/2 inhibition compared with mock control (DMSO). **f**, Mean fold changes between EZHi and mock control derived from RT–qPCR data in **e**. **g**, Top, a schematic outline of the endogenous *GBP1* gene structure in the GBP1–GFP reporter line. Bottom, a schematic of secondary validation for putative regulators of IFNγ transcriptional memory from SGC small-molecule screening in Extended Data Figure 6h,i. **h**, Mean GBP1–GFP fluorescence intensities upon inhibition of putative regulators, measured by FACS. Fluorescence is assessed for mock control (DMSO), naive, priming and reinduction conditions. arb. u., arbitrary units. **i**, Boxplots of the data from **h**, highlighting DOT1Li and EZHi to show individual replicates and signal distribution across the cell population (minimum, first quartile; center line, median; maximum, third quartile). The error bars in **b**, **e** and **h** show the s.e.m. (*n* = 3). The error bars in panels **c** and **f** show the propagated s.e.m. (*n* = 3) between mock and siRNA- or EZHi-treated samples in priming or reinduction. In all panels, *n* values correspond to biological replicates. n.s. > 0.05; •, *P* ≤ 0.05; ••, *P* ≤0.01; •••, *P* ≤ 0.001; ••••, P ≤0.0001. Statistical significance was calculated using the two-sided *t*-test, and prior determination of homo- or heteroscedasticity was done with the *F*-test. Source data

**Extended Data Fig. 8:**
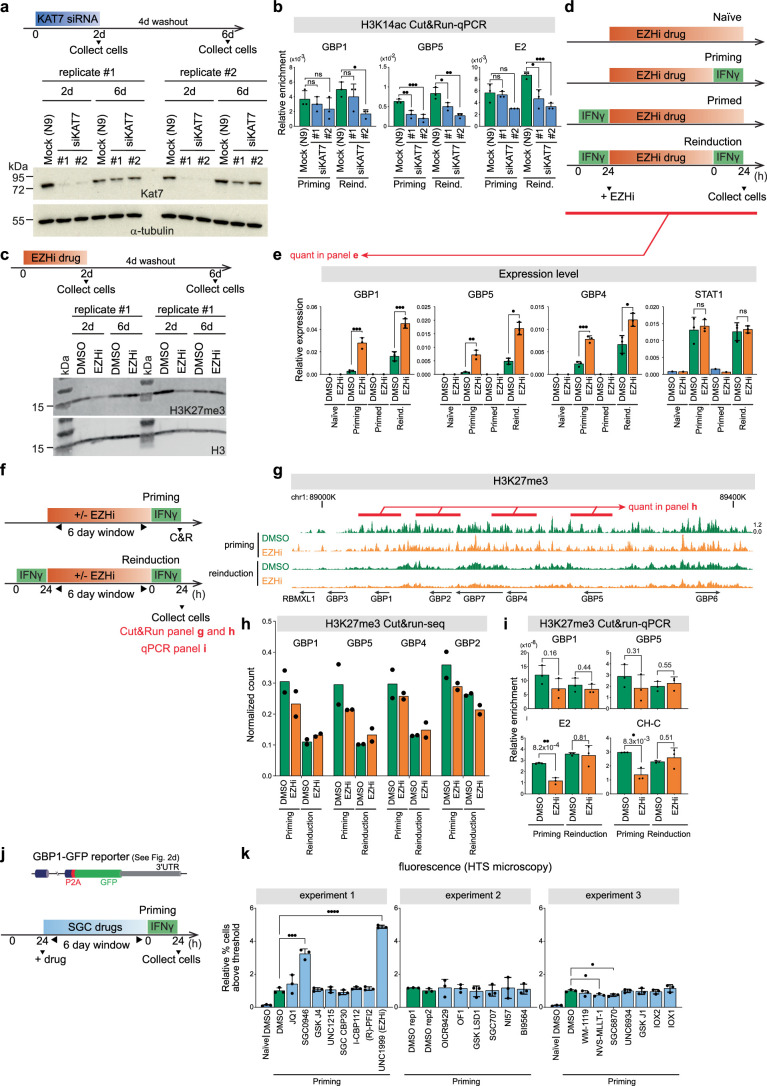
The expression and transcriptional memory of GBP genes is regulated by KAT7, EZH1/2 and DOT1L. **a**, Cells were subjected to Kat7 siRNA treatment (or mock: N9 siRNA) and cell extracts were prepared for SDS-PAGE and immunoblotting, probed with Kat7 antibodies to assess protein level and α-Tubulin antibodies (loading control). **b**, Quantification of H3K14ac Cut&Run-qPCR over indicated loci following IFNγ priming and reinduction following depletion of KAT7 analogous to scheme in Fig. 3a. **c**, Cells were treated with EZH2 inhibitor or DMSO and cell extracts were prepared for SDS-PAGE and immunoblotting, probed with H3K27me3 antibodies. The blots were stripped and assessed for H3 protein (loading control). **d**, Experimental scheme for temporal EZH1/2 inhibition across transcriptional memory regime. **e**, Expression of target genes upon EZH1/2 inhibition compared to DMSO control, across transcriptional memory regime. **f**, Experimental scheme for temporal EZH1/2 inhibition and transcriptional memory regime. **g**, Cut&Run-seq enrichment of H3K27me3 over GBP cluster following EZH1/2 inhibition prior to IFNγ priming and reinduction. Red bars indicate regions over GBP memory genes used for quantification. **h**, Quantification of normalized H3K27me3 Cut&Run sequencing reads over GBP memory genes. Mean and individual data points of two biological replicates are shown. **i**, Quantification of normalized H3K27me3 Cut&Run-qPCR over indicated loci upon mock or EZHi treatment in priming and reinduction conditions. **j**, Top: schematic outline of GBP1-GFP reporter line. Bottom: experimental scheme for screening putative regulators for IFNγ transcriptional memory from SGC small molecule library. **k**, GBP1-GFP fluorescence intensity changes upon temporal drug-based depletion of screened regulators (% cells above the fluorescence threshold normalized to DMSO control in priming condition). Error bars in panels (**d**), (**h**) and (**j**) correspond to SEM (*n* = 3). *n* values for all panels correspond to biological replicates (separate cell populations in tissue culture plates). *P* values for relevant pairwise comparisons are indicated: n.s. > 0.05, • ≤0.05, •• ≤0.01, ••• ≤0.001, •••• ≤0.0001. Statistical significance was calculated with two-sided *t*-test and prior determination of homo- or heteroscedasticity with F-test. Source data

To evaluate the need for active retention of permissive chromatin in transcriptional memory at GBP genes, we focused on the H3K14ac writer KAT7 (also known as MYST2 and HBO1)^24,25^, because this modification is strongly preserved in the primed state following stimulation (Fig. 1c,d). To differentiate the effect on memory from acute effects of IFNγ stimulation, we transiently depleted KAT7 using two distinct short interfering RNAs (siRNAs) (si*KAT7*-1 and si*KAT7*-2) during the memory phase, directly comparing naive and primed cells (Fig. 3a). Immunoblot analysis confirmed that both siRNAs strongly reduced KAT7 levels 2 days after transfection, and that levels recovered by 6 days, the end of the memory window (Extended Data Fig. 8a), thereby minimizing effects on acute transcriptional activation. Transient depletion of KAT7 before IFNγ induction led to a reduction of H3K14 acetylation at key GBP memory genes (Extended Data Fig. 8b), highlighting KAT7’s role in H3K14ac deposition at these genes, consistent with its previously established function as the H3K14 acetyltransferase^25,26^. As expected, KAT7 depletion generally resulted in downregulation of the expression of all tested genes (Fig. 3b,c). However, although the loss of expression of strong memory genes was modest during priming, KAT7 depletion resulted in a stronger downregulation during reinduction. This stronger requirement for KAT7 during reinduction is specific to memory GBP genes; non-memory IFNγ-inducible controls (*STAT1* and *IRF1*) showed a similar reduction in expression in either condition (Fig. 3b,c). Although we cannot exclude the possibility KAT7 has other unknown targets, these results suggest that KAT7-mediated H3K14 acetylation is functionally required to promote GBP memory gene expression, particularly during reinduction. Notably, the selective dependency during reinduction is consistent with the post-stimulus retention of H3K14ac and high enrichment in the primed and reinduction states (Fig. 1c,d).

Next, we targeted EZH1 and EZH2 (EZH1/2), the methyltransferases of the PRC2 complex that generate the repressive H3K27me3 modification^27,28^. To achieve precise temporal control over the complex, we used a selective small-molecule inhibitor of EZH1/2, UNC1999 (referred to hereafter as EZHi)^29^ (Fig. 3d). A 2-day treatment with EZHi led to a significant global reduction of H3K27me3, which recovered by 4 days after inhibitor washout (Extended Data Fig. 8c). EZH1/2 inhibition resulted in the upregulation of all tested genes (Fig. 3e,f), consistent with their known role in gene repression. Of note, the upregulation of GBP memory genes is much stronger during priming than reinduction (Fig. 3e,f and Extended Data Fig. 8d,e). By contrast, the non-memory IFNγ target gene *STAT1* showed no significant upregulation compared with mock in either condition (Fig. 3e,f and Extended Data Fig. 8e). These results indicate that EZH1/2, possibly through their product H3K27me3, repress the expression of GBP memory genes, particularly during priming. This conditional dependency is consistent with the high enrichment of H3K27me3 in the GBP cluster observed in naive cells and during priming. In primed cells and upon reinduction, H3K27me3 is largely depleted, consistent with a minor functional role for EZH1/2 in expression at this stage (Fig. 2b,c). Indeed, analysis of H3K27me3 occupancy across the GBP cluster following EZH1/2 inhibition revealed a mild reduction across GBP genes, particularly during priming, suggesting that levels of this modification might directly affect GBP expression (Extended Data Fig. 8f–i). The collective results from our manipulations of KAT7 and EZH1/2 suggest that both the permissive and repressive chromatin dynamics we observed during priming and memory are functionally required for GBP expression and memory of the primed state.

### Identification of putative regulators of transcriptional memory

To discover other potential regulators of IFNγ priming of GBP genes, we screened a subset of the Epigenetic Chemical Probe Library from the Structural Genomics Consortium (SGC)^30^ containing 21 small molecules targeting chromatin modifiers. To assess these compounds, we treated our GBP–GFP reporter line (Fig. 2d) with a subset of SGC compounds before IFNγ activation of the GBP1 reporter and screened for GFP fluorescence using high-throughput microscopy (Extended Data Fig. 8j,k). Although most compounds did not significantly affect IFNγ induction of GBP1–GFP, we identified SGC0946, a DOT1L (H3K79 methyltransferase)^31^ inhibitor, as a putative hit that leads to enhanced GBP1–GFP expression during IFNγ activation (Extended Data Fig. 8k). We also observed a small but significant downregulation of GBP1–GFP in response to NVS-MLLT-1, an inhibitor of MLLT1 (a chromatin reader component of super elongation complex)^32^, and SGC6870, an inhibitor of PRMT6 (an arginine methyltransferase)^33^. We further identified UNC1999, the EZH1/2 inhibitor, in this screen, validating our RT–qPCR results (Fig. 3e,f and Extended Data Fig. 8e).

We next explored the potential role of the positive hits (the DOT1L inhibitor (DOT1Li) and EZHi) in priming and reinduction. To determine whether the effect of KAT7 depletion on GBP expression described above is specific, we also included an inhibitor for p300 (p300i), an acetyltransferase for H3 and H4 that is distinct from KAT7 (ref. ^34^), and an inhibitor for G9a (G9a-i), a methyltransferase for repressive H3K9 methylation^35^. Our findings revealed that treatments with DOT1Li and EZHi increased GBP1–GFP fluorescence during both priming and reinduction, as measured by fluorescence-activated cell sorting (FACS) (Fig. 3g–i and Extended Data Fig. 7). By contrast, inhibition of G9a did not alter GBP1–GFP expression, suggesting that GBP expression is selectively dependent on Polycomb-mediated repressive chromatin and not G9a-mediated H3K9 methylation (Fig. 3h). Similarly, inhibition of p300 led only to a small, but significant, change in GBP expression, suggesting that histone acetylation mediated by KAT7—rather than p300-mediated acetylation, such as that resulting in H3K27ac—is specifically required for GBP expression (Fig. 3h). Notably, in agreement with our RT–qPCR results (Fig. 3d–f), we observed a higher degree of GBP1–GFP upregulation following EZH1/2 inhibition during priming than during reinduction (Fig. 3h,i), confirming that H3K27me3 and/or EZH1/2 are key limiting factors, particularly during GBP priming.

In summary, these findings suggest that, alongside the repressive role of H3K27me3, DOT1L, or its catalytic products H3K79me1, H3K79me2 and H3K79me3, could contribute to GBP cluster control, warranting future investigation.

### A *cis*-regulatory element controls repression of the GBP cluster

We noted that both permissive and repressive chromatin modifications accumulated not only at GBP genes themselves, but also at intergenic regions across the cluster (Figs. 1 and 2). This suggests that the GBP genes and the surrounding chromatin domain might be regulated globally across the cluster by common control elements.

**Fig. 4:**
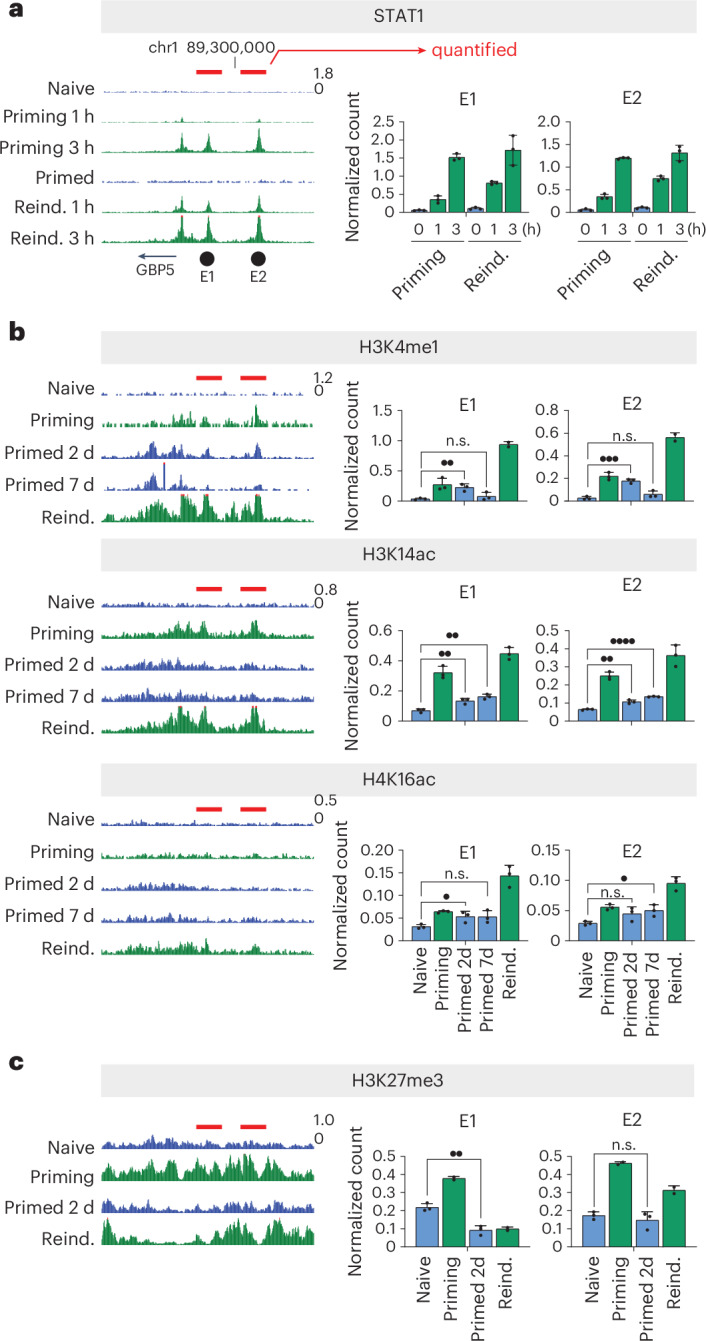
The GBP cluster contains uncharacterized, transcription-factor-bound *cis*-regulatory elements with a transcriptional memory chromatin signature. **a**, Cut&Run-seq of the IFNγ-activated transcription factor STAT1 after 0 h, 1 h and 3 h of activation in naive and primed cells. Genome browser snapshots over the GBP cluster (left) and quantification of normalized Cut&Run sequencing reads over identified *cis*-regulatory elements (right). Data were reanalyzed from ref. ^15^. **b**, Cut&Run-seq enrichment of permissive chromatin modifications during the IFNγ-induced transcriptional memory regime (as shown in Fig. 1), shown as genome browser snapshots over the GBP cluster (left), and quantification of normalized Cut&Run sequencing reads over identified *cis*-regulatory elements (right). **c**, As in **b**, but for the repressive chromatin modification H3K27me3 (*n* = 3 (naive, primed); *n* = 2 (priming, reinduction)). The red bars on all panels indicate the regions used for quantification. Error bars represent the s.e.m. (*n* = 3). *n* values for all panels correspond to biological replicates. n.s. > 0.05; •, *P* ≤ 0.05; ••, *P* ≤0.01; •••, *P* ≤ 0.001; ••••, P ≤0.0001. Statistical significance was calculated using the two-sided *t*-test, and prior determination of homo- or heteroscedasticity was done with the *F*-test. Source data

To discover such elements, we performed unbiased *K*-means clustering of the GBP TAD and its surrounding areas in 10-kilobase (kb) bins. We identified regions with common patterns of enrichment for H3K4me1, H3K14ac, H4K16ac and H3K27me3 during the IFNγ priming and reinduction phases (Extended Data Fig. 4d). Loci with known signatures of heritable chromatin (promoters of *GBP1*, *GBP4* and *GBP5*) clustered together in a distinct group (cluster 4), as determined by principal component analysis (PCA). We identified another PCA cluster (cluster 3) featuring common chromatin signatures that map onto previously identified cohesin-binding sites (cohesin sites B and C^8^) and also to two previously identified *cis*-regulatory elements, E1 and E2 (ref. ^15^), and an uncharacterized locus at the 3′ end of *GBP4*. The E1 and E2 elements, which are 16 and 37 kb upstream of the GBP5 promoter, respectively, are of specific interest given that we have previously found that these were bound by the key IFNγ-induced transcription factor STAT1 (ref. ^15^). In line with the *K*-means clustering analysis, we uncovered an enrichment of permissive chromatin modifications (H3K4me1, H3K14ac and H4K16ac) at these STAT1-bound intergenic E1 and E2 elements (Fig. 4). Similar to GBP gene promoters, these elements also exhibited accumulation of H3K27me3 during priming, which was removed in primed cells (Fig. 4). Overall, these elements and the GBP memory genes generally exhibited similar chromatin landscape dynamics throughout the IFNγ priming process (Figs. 1, 2 and 4).

**Fig. 5:**
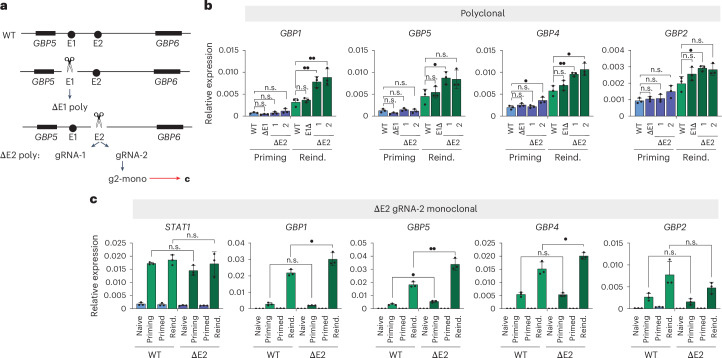
The E2 *cis*-regulatory element controls gene repression across the GBP cluster. **a**, A schematic of the GBP gene cluster, with the indicated CRISPR deletions of E1 and E2, either as a polyclonal (poly) or monoclonal (mono) population. ΔE2 gRNA-1 and ΔE2 gRNA-2 indicate two independent deletions generated with distinct gRNAs (Methods). The monoclonal line was isolated from the ΔE2 gRNA-2 polyclonal line through FACS. **b**, Expression of GBP memory genes in WT and polyclonal ΔE1, ΔE2-1 and ΔE2-2 deletion lines during IFNγ priming or reinduction, measured by RT–qPCR. **c**, Expression of GBP memory genes in WT and monoclonal ΔE2-2 deletion line, measured by RT–qPCR following the IFNγ stimulation regime as in Figure 1a. Error bars represent the s.e.m. (*n* = 3). *n* values for all panels correspond to biological replicates. n.s. > 0.05; •, *P* ≤ 0.05; ••, *P* ≤0.01; •••, *P* ≤ 0.001; ••••, P ≤0.0001. Statistical significance was calculated using the two-sided *t*-test, and prior determination of homo- or heteroscedasticity was done with the *F*-test. Source data

To determine the functional relevance of these putative *cis*-regulatory elements, we generated CRISPR knock-out lines for E1 and E2 and analyzed the consequences for IFNγ priming and reinduction of GBP genes (Fig. 5a). First, we assessed a polyclonal knockout line for E1 and two polyclonal knockout lines for E2, independently generated using different guide RNAs (E2-1 and E2-2, Fig. 5b). Although E1 loss did not have a significant effect on GBP expression, E2-1 and E2-2 showed a marked upregulation of GBP memory genes, primarily upon reinduction (Fig. 5b). To confirm these results and exclude clonal heterogeneity, we subcloned a monoclonal line from the E2-2 population and found that, in agreement with the polyclonal lines, E2 loss results in strong upregulation of memory GBP genes selectively during IFNγ reinduction, but its contribution to initial priming is modest (Fig. 5c). This effect is specific to the strong memory genes; non-memory (*STAT1*) and the weak memory gene were not or were only marginally affected (Fig. 5c).

In summary, we discovered that the E2 *cis*-regulatory element is a transcriptional repressor of GBP genes, not only of the proximal *GBP5* but across the GBP cluster, including distant loci (that is, *GBP1*). Our finding that E2’s role is more pronounced in primed cells than under naive conditions suggests that it has a selective role in transcriptional memory, curbing hyperexpression of GBPs.

### *Cis*-regulatory elements mediate cluster-wide interactions

**Fig. 6:**
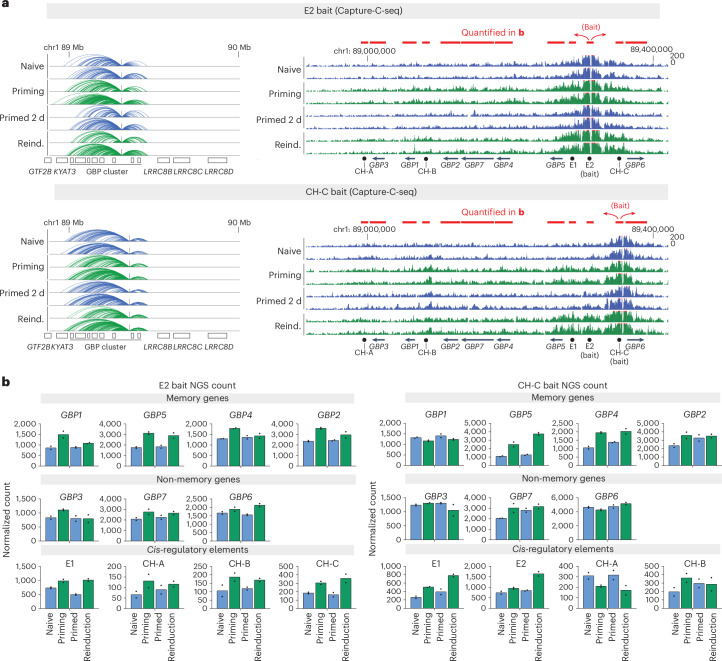
*Cis*-regulatory elements mediate cluster-wide interactions enhanced during IFNγ stimulation. **a**, Capture-C data showing long-range interactions from element E2 (top) or cohesin site CH-C (bottom). The results show zoomed-out (left) and zoomed-in (right) genome browser snapshots from normalized Capture-C sequencing reads at and around the GBP cluster. Genome browser tracks show two biological replicates per condition during the IFNγ-stimulation regime. The red bars correspond to the regions used to measure interactions with the bait locus. **b**, Quantification of normalized Capture-C sequencing reads for E2 (left) or CH-C (right) baits across the GBP cluster: GBP memory genes (*GBP1*, *GBP4*, *GBP5*), GBP non-memory genes (*GBP3*, *GBP6*, *GBP7*, *GBP1P1*) and cis-regulatory elements (E1, E2, cohesin sites (CH-A, CH-B, CH-C)). The experiment was performed in two biological replicates (*n* = 2). Mean and individual data points are shown. Source data

**Extended Data Fig. 9:**
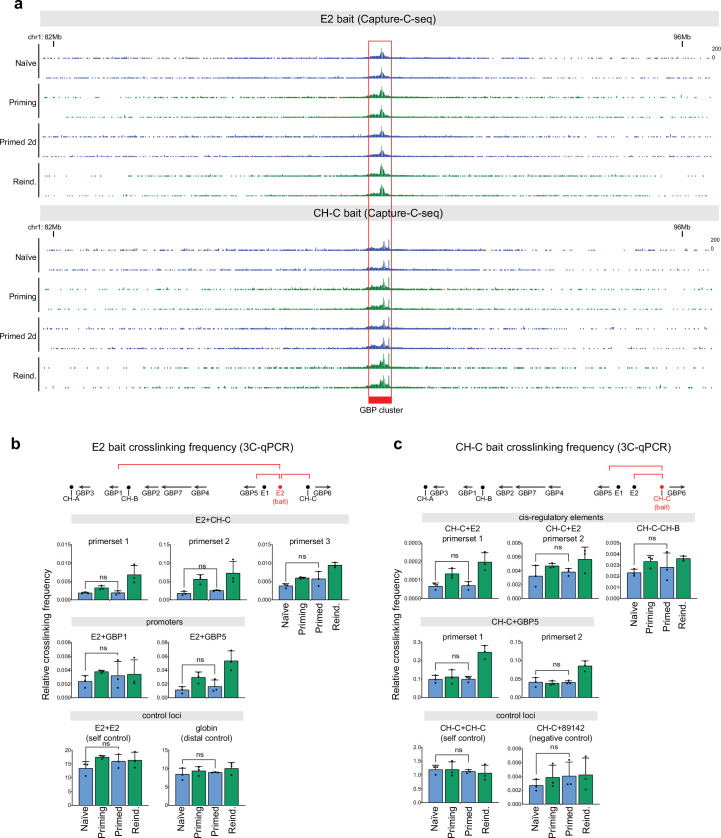
IFNγ induces reciprocal long-range interactions between E2 and CH-C cis-regulatory elements. **a**, Long-range interactions from cis-regulatory elements E2 and CH-C are contained within GBP cluster. The enrichment of long-range interactions from element E2 (top panel) or Cohesin site CH-C (bottom panel) on a multi-megabase scale. The results are presented as genome browser snapshots from normalized Capture-C sequencing reads at and around the GBP cluster. Genome browser tracks show 2 biological replicates per condition in IFNγ-stimulation regime. Red bars indicate the position of GBP cluster. **b**, The interaction level between E2 (bait) and target GBP cluster regions, including CH-C site. The results correspond to relative crosslinking frequency obtained from 3C- quantitative PCR (qPCR) assays. E2 and CH-C interaction is measured by 3 independent qPCR amplicons (E2 + CH-C primerset 1/2/3). The results include control amplicons obtained with primers specific for the intact DNA fragment between DpnII sites after gDNA digestion – E2 + E2 self-control) and a distal control (α-globin cluster). **c**, The interaction level between CH-C (bait) and target GBP cluster regions, including E2 site. The results correspond to the relative crosslinking frequency obtained from 3C-qPCR assay. CH-C and E2 interaction is presented as 2 independent RT-qPCR amplicons (CH-C + E2 primerset 1/2/3), complementing interactions shown in panel **b**. The results include control amplicons: self-control, negative control and distal control (shown in panel **b**). The error bars in panels (**b**) and (**c**) correspond to SEM (*n* = 3). *n* values for all panels correspond to biological replicates. *P* values for relevant pairwise comparisons are indicated: n.s. > 0.05, • ≤0.05, •• ≤0.01, ••• ≤0.001, •••• ≤0.0001. Statistical significance was calculated with two-sided *t*-test and prior determination of homo- or heteroscedasticity with F-test. Source data

Chromatin looping, for example in the context of contacts between enhancers or silencers and gene promoters, has been previously implicated in epigenetic memory^36–39^. To explore this possibility, we used Capture-C^40^, a modified HiC-type chromosome-conformation-capture method that allows for unbiased assessment of all chromatin interactions from a selected genomic viewpoint. We designed probes to isolate the E2 locus as a viewpoint for identifying distal chromatin interactions. We also isolated a cohesin-enriched locus upstream of *GBP6* (cohesin site C, labeled CH-C), which we previously identified as a repressor of the GBP memory genes^8^. Our findings indicate that both E2 and CH-C loci broadly engage chromatin, selectively interacting within the GBP cluster under all conditions (Fig. 6a,b). The boundaries of these interactions coincide with previously identified cohesin-enriched sites^8^ and TAD borders in naive HeLa cells^22^. These interaction boundaries also overlap with the delimited enrichment of heritable chromatin modifications (Figs. 1 and 2 and Extended Data Fig. 3d), suggesting that the GBP cluster forms a specific chromatin domain distinct from neighboring regions. Indeed, we did not detect interactions beyond the GBP locus on chromosome 1, nor *trans*-interactions with other chromosomes (Fig. 6a,b and Extended Data Fig. 9a,b).

**Extended Data Fig. 10:**
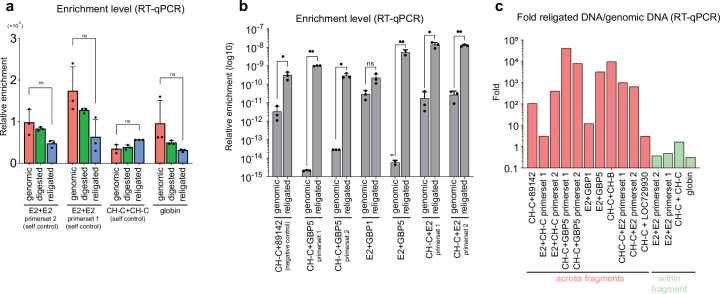
Technical validation of 3C-RT-qPCR assays. **a**, Enrichment level of control amplicons specific for the intact DNA fragment between DpnII sites after gDNA digestion specified in Extended Data Fig. 9. The results correspond to delta-Ct values from qPCR assays in control samples before DNA digestion (genomic DNA), digested DNA and religated DNA. **b**, Enrichment level of selected interaction regions with E2 or CH-C baits specified in Extended Data Fig. 9. The results correspond to delta-Ct values from qPCR assays in control samples before DNA digestion (genomic DNA) and religated DNA on a log(10)-scale. **c**, Assessment of religation efficiency and specificity of primers for target interactions. The results represent fold change between amplification of target regions in religated DNA to genomic DNA. The results correspond to log(10)-transformed delta-delta-Ct values from qPCR assays in religated DNA and genomic DNA. The folds are presented for amplicons used in Extended Data Fig. 9, grouped in categories: across DNA fragments after DpnII digestion (left group) or within a given DNA fragment after DpnII digestion (right group). The error bars in panels (**a**) and (**b**) correspond to SEM (*n* = 3). *n* values for all panels correspond to biological replicates (separate cell populations in tissue culture plates). *P* values for relevant pairwise comparisons are indicated: n.s. > 0.05, • ≤0.05, •• ≤0.01, ••• ≤0.001, •••• ≤0.0001. Statistical significance was calculated with two-sided *t*-test and prior determination of homo- or heteroscedasticity with F-test. Source data

Although interactions within the GBP cluster occur in the absence of GBP gene expression (naive cells), these interactions are markedly enhanced by IFNγ priming (Fig. 6a,b). This observation suggests that IFNγ induces compaction of the cluster, analogous to an earlier report of long-range compaction of the GBP cluster in mouse bone-marrow-derived macrophages in response to either type 1 IFN or IFNγ^41^. Both the E2 and CH-C loci exhibited enhanced engagement with virtually all genes and loci tested in the cluster. However, these contacts were transient during IFNγ activation and were reset in primed cells (Fig. 6c,d). We did not find any memory of long-range contacts that would maintain the same level of engagement between E2 and CH-C with the GBP cluster upon reinduction. We validated these Capture-C results by conventional 3C–qPCR experiments (Extended Data Fig. 9c,d and Extended Data Fig. 10). Notably, in addition to contacts between GBP genes, we also identified increased interactions between the E2 and CH loci (Extended Data Fig. 9c,d), suggesting that both repressive *cis*-regulatory elements could regulate each other.

### Delayed activation of repressive elements facilitates GBP hyperexpression

**Fig. 7:**
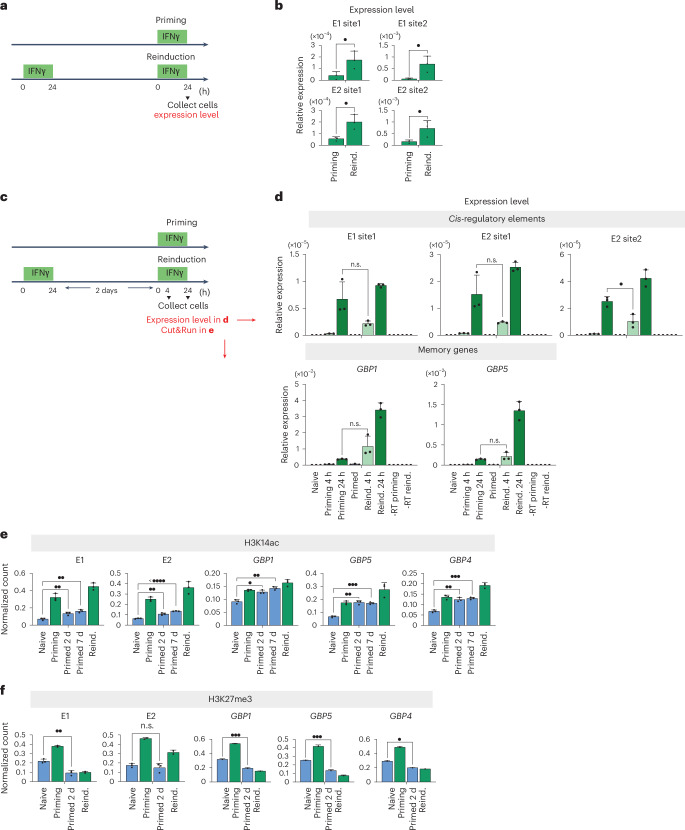
Delayed activation of *cis*-regulatory elements facilitates hyperactivation of GBP memory genes following IFNγ priming. **a**, The experimental regime outlining the timing of IFNγ-incubation and cell collection**. b**, Expression levels of the target *cis*-regulatory elements E1 and E2, assessed by RT–qPCR in IFNγ priming and reinduction conditions. Expression levels of two independent amplicons per *cis*-regulatory element are shown. **c**, The experimental regime outlining the timing of IFNγ incubation, cell collection and downstream analysis for experiments in **d** and **e**. **d**, Expression levels of target loci (*cis*-regulatory elements, GBP memory genes and control non-memory genes), measured by RT–qPCR following the IFNγ-stimulation regime. cDNA synthesis negative controls (−RT, no reverse transcriptase) are included for priming and reinduction conditions. **e**,**f**, A comparative quantification of H3K14ac (**e**) and H3K27me3 (**f**) enrichment between *cis*-regulatory elements and GBP memory genes. The results correspond to the Cut&Run sequencing read counts presented in Figures 1, 2 and 4. The error bars in panels **b**, **d** and **e** correspond to the s.e.m. (*n* = 3). n.s. > 0.05; •, *P* ≤ 0.05; ••, *P* ≤0.01; •••, *P* ≤ 0.001; ••••, P ≤0.0001. Statistical significance was calculated using the two-sided *t*-test, and prior determination of homo- or heteroscedasticity was done with the *F*-test. Source data

We next explored how the GBP cluster can be strongly activated by IFNγ, despite the repressive influence of the E2 element. Considering that E1 and E2 are bound by the STAT1 transcription factor (Fig. 4), we expected that these elements would produce non-coding RNAs, as is typical for enhancer elements^42^. Indeed, we detected non-coding RNAs by RT–qPCR specifically upon IFNγ activation and used these as a readout of the activity of these elements. Notably, both *cis*-regulatory elements showed hyperactivation during IFNγ reinduction compared with priming (Fig. 7a,b), indicating they exhibit transcriptional memory, similar to GBP memory genes.

We hypothesized that the GBP genes and the E1 and E2 elements are activated with different kinetics, allowing GBP genes to be rapidly activated but their expression to be curbed at a later stage. To test this idea, we measured the expression of both E1 and E2, as well as GBP genes, at early (4 h) and later (24 h) timepoints (Fig. 7c). We found a striking difference in activation dynamics: the GBP memory genes, which are poorly expressed during priming, show very rapid activation upon reinduction (Fig. 7d). By 4 h of IFNγ exposure, primed cells showed a higher expression than at any time during priming. By contrast, the critical E2 element (as well as E1, to a lesser extent) showed a marked delay in reactivation. At 4 h of reactivation, both E1 and E2 were expressed at levels much lower than their priming levels (Fig. 7d). These results indicate that the *cis*-regulatory elements exhibit a delay of IFNγ-mediated transcriptional activation compared with GBP memory genes.

Interestingly, analysis of chromatin signatures revealed that the *cis*-regulatory elements exhibit a marked loss of H3K14ac relative to the levels established during priming, whereas GBP genes maintain these marks to levels similar or even higher than those established during IFNγ priming (Fig. 7e), consistent with their more rapid reactivation upon IFNγ reinduction. H3K27me3 was lost from both E1 and GBP genes in primed cells and upon reinduction. However, we noticed that the E2 element does not show such a pronounced loss of H3K27me3 (Fig. 7f), suggesting that E2 maintains a more repressive chromatin signature.

Overall, the delayed expression of the E1 and E2 elements, weaker maintenance of permissive chromatin marks and the retention of repressive H3K27me3 at E2 could explain how GBP memory genes can initially ‘escape’ from E2’s repressive function. We hypothesize that, at a later stage during IFNγ re-exposure, the E2 element curbs GBP activation, preventing excessive hyperactivation. This model is consistent with the expression dynamics detected in our single-cell GBP1–GFP expression analysis (Fig. 2d,e). We observed that the number of cells expressing GBP1 during priming gradually increased over 24 h. By contrast, in primed cells, the number of cells that hyperactivate GBP1 initially increased but plateaued after approximately 10 h (Fig. 2e). This temporal dynamic is consistent with the expression dynamics and chromatin status of the repressive *cis*-regulatory element in the GBP cluster, whose delayed activation could allow initial strong GBP induction while preventing excessive activation.

## Discussion

In mammals, the heritable priming of cells by IFNγ has been shown to last for several weeks through multiple cycles of cell division, in the absence of ongoing transcription^5,7,8^. Despite its strong epigenetic nature, the molecular basis for what carries this memory has remained elusive.

In this study, we identified: (1) selective maintenance of permissive chromatin marks and depletion of repressive marks in IFNγ-primed cells; (2) a functional role for KAT7 and EZH1/2 in memory; and (3) a local *cis*-acting element that represses the GBP cluster. The stable maintenance of permissive chromatin modifications (H3K4me1, H3K14ac and H4K16ac) in the GBP-cluster in the absence of IFNγ suggests that these modifications can actively propagate in proliferating cells, even without the initial trigger. Read–write mechanisms that engage in a feedback loop have been described for repressive modifications, such as Polycomb-mediated H3K27me3 (refs. ^43,44^) and H3K9 methylation at heterochromatin^45^, as well as for DNA CpG methylation^46,47^. Specific permissive chromatin modifications, including those identified in this work, have been shown to be locally maintained on mitotic chromosomes, constituting ‘mitotic bookmarks’^48–50^. This behavior is consistent with a role as a mediator of transcriptional memory. However, how they engage in read–write feedback to avoid dilution during cell division remains unclear and is an important future direction of inquiry. Furthermore, our results are consistent with the previously identified role of H3K4me1 in enhancer priming^51–53^, indicating that this chromatin modification can be stably maintained. We previously discovered that the transcription factors IRF1 and STAT1, which are essential for GBP gene expression, target the promoters of GBP genes earlier and faster in primed cells, correlating with enhanced reactivation. We speculate that the mitotically inherited permissive chromatin state could facilitate enhanced recruitment of STAT1 and IRF1, resulting in enhanced reactivation of GBP genes.

Our findings reveal that, alongside maintaining permissive chromatin, H3K27me3, which is regulated by PRC2, is established across the GBP gene cluster during priming. This occurs even in cells in which GBP genes are expressed and is essential to suppress GBP expression. This response could be part of a mechanism to limit the otherwise strong and rapid activation of IFNγ targets. This is consistent with our finding that limiting PRC2 activity (the H3K27me3 methyltransferase complex), results in GBP hyperactivation. Notably, H3K27me3 repressive chromatin is selectively depleted from memory genes and maintained at a low level in primed cells. The inability to re-establish H3K27me3 following IFNγ priming could be important in the priming of GBP genes. Furthermore, we showed that IFNγ exposure alters H3K27me3 and H3K14ac enrichment at GBP genes, irrespective of their expression levels. This suggests that transcription itself might not directly induce the primed state. Instead, other IFNγ-activated factors may regulate quantitative changes in the chromatin status that in turn increase the probability of future gene reactivation. The spatial compaction of the GBP cluster during IFNγ gene activation is not inherited but could further facilitate robust activation. For instance, promoters in the GBP cluster might also act as enhancers for neighboring GBP genes (Epromoters), as has been reported for type I IFN targets in K-562 lymphoblast cells^54^.

Of note, we discovered that the E2 element exerts a repressive effect on GBP expression, particularly during extended exposure to IFNγ reactivation. The E2 element has the signatures of an enhancer that is bound by the STAT1 transcription factor and generates RNAs during activation; however, in the context of GBP expression, it acts as a repressive element. How an element with features of an enhancer acts as a repressive element is unclear, although not unprecedented because enhancer elements have been reported to act as silencers in specific contexts^55^, with some acting as bifunctional elements depending on the local context^56^. E2 might function as a transcription-factor sink, competing with promoters. Alternatively, it could act in conjunction with cohesin-binding sites at the GBP cluster boundaries, which we have previously shown to restrict GBP cluster expression^8^. How these elements exert their repressive effect on GBP expression remains an open question.

**Fig. 8:**
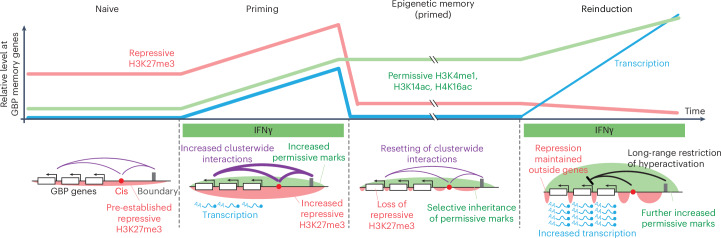
The proposed model for IFNγ-inducible chromatin-based transcriptional memory at GBP genes. The GBP cluster is embedded in a broad domain of low-level repressive H3K27me3 chromatin. IFNγ activation results in GBP transcription, and increased long-range interactions between the *cis*-regulatory elements, cluster boundaries and genes. It further results in establishing activating chromatin in part by KAT7, but also a further elevation of repressive chromatin mediated by PRC2. In the primed state, transcription is lost but permissive chromatin is selectively retained and mitotically heritable while suppressive H3K27me3 chromatin is locally depleted from GBP genes. This allows for rapid, strong reactivation of GBP genes upon re-exposure to IFNγ. The *cis*-regulatory element acts to repress GBPs across the cluster preventing hyperactivation by IFNγ.

In summary, our findings suggest that transcriptional memory is mediated by a balance of unique permissive and repressive chromatin modifications that are differentially inherited in IFNγ-primed cells, resulting in memory of prior IFNγ exposure (Fig. 8). Interferons are important mediators of innate and adaptive immunity and are central to the priming of innate immune cells^57^. Key effectors of interferon-γ signaling, such as macrophages^57^, play a role in trained immunity, in which the organisms maintain a long-term memory of prior immune activation^58^. The mechanisms uncovered here might contribute to the cellular memory of innate immune signals that underpin the physiological state of trained immunity.

## Methods

### Cell culture

HeLa Kyoto cells (female, RRID: CVCL_1922) were grown in Dulbecco’s modified eagle medium (DMEM) containing high glucose and pyruvate (Thermo Fisher Scientific, cat. no. 41966-029) supplemented with 10% NCS (newborn calf serum, Thermo Fisher Scientific, cat. no. 16010-159) and 1% penicillin–streptomycin (Thermo Fisher Scientific, cat. no. 15140-122) at 37 °C, 5% CO_2_. For temporal depletion experiments using siRNAs or drugs, 1% penicillin–streptomycin was not included in the DMEM. Primary neonatal fibroblasts (Lonza, cat. no. CC-2509) were grown in DMEM containing glucose, glutamine and pyruvate (Gibco) supplemented with 10% fetal bovine serum (FBS) (Gibco). THP-1 cells (ATCC, cat. no. TIB-202) were grown in Roswell Park Memorial Institute (RPMI) 1640 medium containing GlutaMAX (Gibco) supplemented with 10% FBS (Gibco), 10 mM HEPES and 1.0 mM sodium pyruvate (Gibco). For passaging, cells were washed with 1× DPBS (Thermo Fisher Scientific), detached with TrypLE Express phenol red (Thermo Fisher Scientific) and resuspended in DMEM. THP-1 cells were collected by centrifugation at 500*g* for 5 min. Cells were counted using Countess Cell Counting (Thermo Fisher Scientific). Transfection of cells was performed using Lipofectamine LTX (Thermo Fisher Scientific). Cells were routinely tested for mycoplasma contamination.

### Transcriptional memory assay

Cells were primed with 50 ng ml^–1^ IFNγ (Merck) or left untreated for 24 h; this was followed by IFNγ washout with DPBS (ThermoFisher) and trypsinization by TrypLE (Thermo Fisher Scientific) to collect cells. Cells were cultured with fresh medium for another 48 h, unless stated otherwise. Next, naive and primed cells were induced by IFNγ for 24 h. After 24 h, cells were trypsinized and collected, and the pellets were processed for subsequent experiments.

### Cut&Run

Cut&Run was performed using CUTANA v3 kit (Epicypher) with mild cross-linking (1 min incubation with 1% formaldehyde at room temperature). The antibodies used targeted H3K4me1 (Epicypher, cat. no. 13-0057), H3K4me3 (Epicypher, cat. no. 13-0041), H3K14ac (Merck, cat. no. 07-353), H4K16ac (Merck, cat. no. 07-329) or H3K27me3 (Cell Signaling Technology, cat. no. 9733). The experiments were performed in biological duplicates or triplicates. For Cut&Run–qPCR, the samples were adjusted to a fixed concentration of 0.02 ng µl^–1^, and 0.1 ng was used per reaction for RT–qPCR. The primers are listed in Supplementary Table 1. The qPCR protocol consisted of an initial cycle at 95 °C for 3 min, followed by 50 cycles of 95 °C for 10 s and 60 °C for 30 s; qPCR was followed by a melting curve step (temperature range, 95–60 °C). Data were normalized to total recovered DNA per sample. Alternatively, for experiments in which the DNA yield was sufficient (Fig. 2i), 10% input was generated and used for normalization of sample data, according to published protocols (Cell Signaling Technology, cat. no. 86652). For Cut&Run-seq, the sequencing libraries were performed with NEBNext Ultra II DNA Library Prep Kit for Illumina (NEB), according to the published protocol^59^. The samples were multiplexed using NEBNext Multiplex Oligos for Illumina (Index Primers Set 1 and 2) (NEB). Size selection was performed using Ampure XP beads (Beckman Coulter) and adjusted for nucleosomal DNA fragment size (150 bp, excluding adapters). The yield and quality of sequencing libraries were assessed using the Qubit HS dsDNA Quantification Assay Kit (Thermo Fisher Scientific) and TapeStation 4150 System (Agilent). Multiplexed libraries were diluted to concentrations of 1, 2 or 4 nM and sequenced on the NextSeq 550 (Illumina) with the NextSeq 500/550 High Output v2.5 (75 cycles PE) kit (Illumina).

### Expression (RT–qPCR)

Cell pellets (1 million cells per sample) were re-suspended in 0.2 ml PBS and 0.8 ml TRIzol Reagent (Thermo Fisher Scientific). Cells were lysed by vortexing and incubated for 5 min at room temperature. Next, 0.16 ml chloroform was added per sample, mixed and incubated for 5 min at room temperature, followed by centrifugation at 12,000*g* for 15 min at room temperature. The aqueous phase was mixed 1:1 (vol/vol) with 100% isopropanol and incubated at –20 °C for 30 min, followed by centrifugation at 12,000*g* for 30 min at 4 °C. The supernatant was removed and the pellet was washed with 1 ml of 75% ethanol and air-dried for 10 min. RNA pellets were re-suspended in 50 μl nuclease-free water. Any residual DNA contamination was removed using the TURBO DNA-free Kit (Thermo Fisher Scientific). For cDNA synthesis, 1.5–2 µg RNA per sample was used with the High-Capacity RNA-to-cDNA Kit (Applied Biosystems). Final cDNA samples were diluted ten times before qPCR measurements. The qPCR assay was performed with iTaq Universal SYBR Green Supermix (Biorad). The primers are listed in Supplementary Table 1. Primer efficiency was determined computationally from amplification efficiency per PCR cycle using LinReg software^60^. The qPCR protocol consisted of an initial cycle of 95 °C for 3 min, followed by 50 cycles of 95 °C for 10 s and 60 °C for 30 s. This was followed by a melting curve step (temperature range, 95–60 °C). The relative expression level of target genes was calculated using the efficiency-corrected 2^–ΔΔCt^ method^61^ with normalization to β-actin.

### RNA interference and small-molecule inhibitors

#### siRNA

All siRNA transfections were performed on trypsinized cells. Cells were seeded in 6-well plates at a density of 2.25 × 10^5^ cells per well and were supplemented with 5 nM siRNA premixed with Opti-MEM Reduced Serum Medium (Gibco) and Lipofectamine RNAi Max Transfection Reagent (Thermo Fisher Scientific). The siRNAs used included si*KAT7*-1 (cat. no. 108177) and si*KAT7*-2 (cat. no. 108179), both obtained from Silencer Select Pre-Designed and Validated siRNA (Thermo Fisher Scientific). As a control, we used Neg9 (N9)-depleting siRNA targeting 5′-UACGACCGGUCUAUCGUAGTT-3′.

#### Small-molecule inhibitors (EZHi and DOT1Li)

EZHi (UNC1999) and DOT1Li (SGC0946) were obtained from the Structural Genomics Consortium (SGC)^30^. Inhibitor incubation was conducted on trypsinized cells. For the FACS experiments, GBP1–GFP-expressing cells were seeded in 24-well plates at a density of 1.6 × 10^4^ cells per well in 1 ml DMEM supplemented with 2 μM of the respective inhibitor. For the Cut&Run-seq and Cut&Run-qPCR experiments, cells were seeded in 6-well plates at a density of 2.25 × 10^5^ cells per well in 2 ml DMEM supplemented with 2 μM of the respective inhibitor. Each experiment included a mock control with cells supplemented with 100% DMSO at the same volume as the inhibitors. Cells were washed with PBS, trypsinized, either collected as a pellet or cross-linked (in 1% formaldehyde for 10 min on a rotator at room temperature) and quenched with 0.25 M glycine. The cells were then subjected to FACS, as described below.

### Immunoblot analysis

Cell pellets were resuspended in 200 µl lysis buffer (10 mM Hepes, pH 7.9; 1.5 mM MgCl_2_; 10 mM KCl; 500 nM DTT; 1× cOmplete, EDTA-free Protease Inhibitor Cocktail). The mixture was incubated for 15 min on ice, followed by 5 min of centrifugation (4 °C, 700*g*). For H3K27me3 analysis, this process was followed by nuclear extraction in 60 µl lysis buffer supplemented with 0.1% NP40 (IGEPAL) for 15 min on ice, followed by centrifugation for 5 min (4 °C, 1,000*g*). For all samples, the pellet was resuspended in 40 µl RIPA buffer (10% glycerol; 10 mM Tris-HCl, pH 7.5; 150 mM NaCl; 1% NP40; 1% DOC; 0.1% SDS; 1 mM DTT), incubated 20 min on ice and centrifuged for 20 min (4 °C, 12,000*g*). Protein concentration in the supernatant was assessed using the Bradford assay, and 10 µg of total protein was resuspended in 10 µl RIPA buffer mixed with 10 µl 2× protein sample buffer (250 mM Tris–HCl, pH 6.8; 20% glycerol; 2% SDS; 0.4% (wt/vol) Orange G; 10% β‐mercaptoethanol) and incubated at 98 °C for 5 min. Samples were separated on a 10% or 12% SDS–PAGE gel (BioRad), then transferred to nitrocellulose membranes (BioRad Transblot Turbo), blocked with either Intercept (PBS) Blocking Buffers (LI‐COR) or 5% milk (1× PBS, 0.05% Tween-20, 5% (wt/vol) skim milk powder) for 1 h and incubated overnight with primary antibodies at 4 °C. The next day, blots were washed three times with TBST (20 mM Tris–HCl, pH 7.5; 150 mM NaCl; 0.1% Tween‐20) and incubated with secondary antibodies for 1 h, followed by three TBST washes. The blots were analyzed using the Odyssey Imaging System (LI‐COR) or exposed to photosensitive film, followed by standard development and fixation. The antibodies used targeted KAT7 (Abcam, cat. no. ab190908, rabbit monoclonal), α-tubulin (Sigma, cat. no. T9026, mouse monoclonal), H3K27me3 (Cell Signaling, cat. no. C36B11, rabbit monoclonal) or H3 (Abcam, cat. no. ab1791, rabbit polyclonal).

### GBP1–GFP line generation

The GBP1–GFP HeLa cell line was created using the LentiCRISPR V2-Blast (Addgene, cat. no. 83480) vector containing Cas9 sequence and a gRNA targeting exon 11 of *GBP1*, which encodes the stop codon (see Supplementary Table 1 for gRNA sequence). The homology repair template cloned in pUC19 consisted of a synthesized P2A-GFP cassette (Life Technologies) flanked by the GBP1 homology arms that match coordinates chr1: 89053857–89053357 and 89053357–89052857. A silent mutation in the protospacer-adjacent motif (PAM) recognition sequence was introduced in the gRNA target of the homology arm to prevent Cas9 recutting after successful repair. The homology repair template was linearized before reverse cotransfection with the plasmid containing the Cas9 and gRNA (in a 1:3 ratio) using Lipofectamine 3000 (Thermo Fisher Scientific). The following day, cells were treated with blasticidin for 48 h. Subsequently, cells were induced with IFNγ for 24 h and sorted to single cells by FACS on the basis of GFP fluorescence to generate monoclonal lines. Cells were maintained in culture for at least 2 weeks to eliminate the effects of IFNγ priming before being used in experiments.

### High-content microscopy screening of small molecules

GBP1–GFP cells were seeded in 96-well plates (1.6 ×10^4^ cells per well) in 0.2 ml (DMEM), supplemented with 2 μM of the respective inhibitor (described above). A mock control, consisting of cells supplemented with 100% DMSO at the same volume as the inhibitors, was included in each experiment. To minimize temperature effects on the readout, border wells of the plate were filled with PBS and excluded from analysis. Following transfection, the plates were incubated at room temperature for 45 min before transitioning to standard growth conditions. For collection, the cells were washed with PBS, cross-linked (1% formaldehyde, 10 min on rotator at room temperature) and quenched with 0.25 M glycine. Next, the cells were washed with PBS and stored at 4 °C for up to 7 days before imaging with the Opera Phenix Plus High-Content Screening System (Perkin Elmer). GFP fluorescence thresholds were adjusted per plate on the basis of the (non-fluorescent) and priming (fluorescent) conditions. The final threshold per plate was selected on the basis of the *Z* score between conditions. The percentage of cells above the threshold was used to compare controls and inhibitor-treated samples, enabling the identification of hits that affected GBP1–GFP expression.

### Live-cell imaging

GBP1–GFP reporter cells (described above) were transduced with a pBABE retrovirus expressing H2B-mRFP^62^ to mark nuclei for analysis. Clones were selected by puromycin resistance and scored for robust H2B-mRFP expression. Cells were primed following the procedure described in ‘Transcriptional memory assay.’ Five days after IFNγ washout (the memory window), cells were transferred into the chambers of a µ-Dish 35 mm Quad dish (Ibidi) with a polymer coverslip and were cultured for 24 h in CO_2_-independent Live Cell Imaging Solution (Invitrogen) supplemented with 10% FBS (Life Technologies). Cells were then either induced with 50 ng ml^–1^ IFNγ or left untreated and imaged at 1-h intervals for 24 h, starting 40–60 min after IFNγ was added. Cells were imaged using a temperature-controlled Leica DMI6000 widefield microscope at 37 °C, equipped with a Hamamatsu Flash Orca 4.0 sCMOS camera, using a ×40 1.4-numerical-aperture objective (HC PLAN APO). GFP fluorescence was quantified on the basis of nuclei detection using TrackMate Cellpose plugin for ImageJ. The time-lapse tracks were analyzed in R and filtered to include only continuous tracks lasting at least 24 h. Each track was then normalized on the basis of the first three time points. Data points from cells transitioning through mitosis were excluded owing to a temporary spike in background fluorescence. The resulting tracks were used to determine the cut-off value for cells with GBP1–GFP expression or hyperactivated expression. To create a stringent cut-off, cells were deemed to express GBP1–GFP if their fluorescence intensity was at least three times the interquartile range above the third quartile of the GFP signal in naive cells. Similarly, hyperactivated expression was defined as GFP fluorescence being at least three times the interquartile range above the third quartile of the GFP signal in cells during priming. The intensities below and above said thresholds were determined separately for each time point and biological replicate. The average intensities of biological replicates per time point were used to derive the fraction of cells with a given GBP1–GFP expression status.

### FACS

For FACS and cytometry, cells were collected by centrifugation for 5 min at 500*g*, resuspended in ice-cold sorting medium (1% FBS in PBS, 0.25 mg ml^–1^ amphotericin B (Thermo Fisher Scientific), 0.25 μg ml^–1^/10 μg ml^–1^ amphotericin B/gentamicin (Gibco)) and filtered using 5-ml polystyrene round-bottom tubes with cell-strainer caps (Falcon) before sorting with a FACSAria III Cell Sorter (BD Biosciences). After sorting, the cells were collected in 96-well plates containing conditional medium (a 1:1 mixture of fresh complete medium and medium collected from proliferating cell cultures passed through a 0.45-μm filter, supplemented with 20% FBS, 0.25 mg ml^–1^ amphotericin B (Thermo Fisher Scientific) and 0.25 μg ml^–1^/10 μg ml^–1^ amphotericin B/gentamicin (Gibco)). The purity, gating parameters and fluorescence threshold for GBP1–GFP samples were assessed separately in each FACS experiment by cross-comparison of samples to negative and positive controls—DMSO-treated non-IFNγ-induced or IFNγ-reinduced samples, respectively.The cell population counts for each sample across the experiments and samples was within 13,441–23,927 cells. After IFNγ induction, 50 × 10^6^–80 × 10^6^ cells were collected in sorting medium per biological replicate, which were then sorted into 15-ml Falcon tubes on the basis of GFP signal, using the non-induced cells for gating. Each replicate after sorting contained 5 × 10^5^ (Fig. 2j,k and Extended Data Figs. 8f–i) or 1 × 10^6^ (Fig. 2i) cells. The cells were pelleted and either seeded in complete medium for growth and future collection or cross-linked for 1 min at room temperature using 1% formaldehyde for subsequent Cut&Run analysis.

### CRISPR–Cas9 cloning and genome engineering

E1, E2-1 and E2-2 mutants were generated using CRISPR–Cas9 technology as double-cut *cis*-regulatory elements’ deletion lines. The gRNAs were designed using IDT and CRISPick (Broad Institute) tools. The gRNA sequences are specified in Supplementary Table 1. Relevant gRNA pairs were cloned into lentiCRISPR v2-Blast (Addgene, cat. no. 83480) and lentiCRISPR v2 (Addgene, cat. no. 52961), allowing for dual antibiotic resistance after transfection (blasticidin and puromycin, respectively).

Resultant plasmids were transfected into HEK293T cells using viral packaging plasmid psPAX2 (Addgene, cat. no. 12260) and viral envelope plasmid pMD2.G (Addgene, cat. no. 12259) at a molar ratio of 4:3:1, respectively, followed by incubation at 37 °C for 3 days. Culture medium containing lentiviral particles was collected, filtered through 0.45-µm filters, incubated with 8 mg ml^–1^ Polybrene Reagent (Merck) for 1 h, mixed 1:1 with fresh medium and added to HeLa cells for transduction. Two days after transduction, cells were selected with 5 mg ml^–1^ blasticidin and 1 mg ml^–1^ puromycin. Mutant lines were collected and validated using gDNA PCR and Sanger sequencing using the oligonucleotides specified in Supplementary Table 1. The E2-2 monoclonal line was generated by single-cell FACS, as described above.

### Genome architecture (Capture-C-seq and 3C–qPCR)

Capture-C-seq was performed as published^40^, with the following specifications. The viewpoints were selected and their specific probes were designed using Capsequm2 software. For each sample, 5 × 10^6^ cells were used. DNA was digested with DpnII restriction enzyme (NEB) and religated with the T4 DNA HC ligase (Thermo Fisher Scientific). Sonication was performed on Q500 machine (QSonica) to obtain ~200-base-pair DNA fragments, following preoptimization of sonication conditions on genomic DNA control samples. Sequencing libraries were synthesized and multiplexed with NEBNext Ultra II kit (NEB). Ampure XP (Beckman Coulter) was used for size selection. The experiment was adapted for high-specificity sequencing (double hybridization with probe titration). The hybridization was performed in two separate pools with 5′-biotinylated oligonucleotides for either the E2 or CH-C viewpoint. The oligonucleotides are listed in Supplementary Table 1. Each pool’s experiments were performed in biological duplicate. Sequencing was performed on NextSeq550 sequencer (Illumina) using the NextSeq 500/550 High Output Kit v2.5 (75 Cycles PE) (Illumina).

3C–qPCR was performed according to the published protocol^63^ with the following specifications. For each sample, 0.8 × 10^6^–1 ×10^6^ cells were used. DNA digestion was performed using a DpnII restriction enzyme (NEB), and subsequent religation was done using T4 DNA HC ligase (Thermo Fisher Scientific). Residual proteins and RNA were removed using Proteinase K (Ambion) and RNase A (Thermo Fisher Scientific), following the manufacturer’s protocols. DNA was purified with phenol-chloroform-isoamyl alcohol and ethanol, according to the published protocol^64^. Sample yield and quality were assessed through gel electrophoresis. Qubit BR dsDNA assays (Thermo Fisher Scientific) and RT–qPCR analyses were performed on genomic, digested and re-ligated controls. For RT–qPCR assays, each final 3C sample was diluted to 25 ng DNA per reaction. Ct values were normalized according to the published protocol^63^. LinReg software^60^ was used to determine primer efficiency from amplification efficiency per PCR cycle, and amplification of E2 or CH-C baits in the digested fragment served as a loading control. The oligonucleotides used for RT–qPCR are listed in Supplementary Table 1.

### Bioinformatic data analysis and statistics

#### Unix

All Unix commands were performed in conda environments. For Cut&Run-seq data analysis, raw reads (FASTQ) for each experiment were downloaded from Basespace servers (Illumina) using basespace-cli and concatenated per sample using base Unix. Read quality was assessed using Basespace (Illumina) and FastQC^65^ software. Next, reads were mapped to the hg38 genome with Bowtie2 (ref. ^66^), with trimming conditions adjusted depending on read quality. SAM to BAM conversion, BAM sorting and indexing were performed using Samtools v1.1 (ref. ^67^). Read duplicates were removed using Picard (MarkDuplicates command)^68^. Sorted and duplicate-removed BAM files underwent read count, normalization to counts per million mapped reads (CPM) and conversion to bigwig format using Deeptools v2 (bamcoverage command)^69^. Bigwig files were visualized with the Integrative Genomics Viewer (IGV)^70^ and WashU Epigenome Browser^71^. The read count matrices for cross-comparison between conditions and samples were generated using Deeptools v2 (multibigwigsummary command)^69^.

For Capture-C-seq analysis, raw reads (FASTQ) for each experiment were downloaded from Basespace servers (Illumina) using basespace-cli and concatenated per sample with base bash. Read quality was assessed using Basespace (Illumina) and FastQC^65^ software. Next, reads were processed with the CapCruncher pipeline^72^, up to the generation of compressed contact matrices (HDF5 format). Contact matrices were further processed and converted to bedpe format using the Cooler tool^73^. Filtering and normalization were performed using custom-made scripts in base Unix, adhering to the published protocol^72^. Final bedpe or bedgraph files were visualized using the IGV^70^ and WashU Epigenome Browser^71^. The read count matrices for cross-comparison between conditions and samples were generated with Deeptools v2 (multibigwigsummary command)^69^.

#### R

The read count matrices (Deeptools v2 multibigwigsummary ouput) from Cut&Run-seq and Capture-C-seq were processed in R v4.1 (ref. ^74^) with the RStudio v1.4 (ref. ^75^) interface. The matrices were annotated, filtered (removal of 0 count reads) and quantified (mean, standard error, folds between conditions and statistics) using custom-made scripts. Data wrangling was performed using base R and the dplyr package^76^. Data visualization was done using the ggplot2 (ref. ^77^) and ggrepel^78^ packages.

Venn diagrams were created using the VennDiagram package^79^. To exclude background noise and lowly enriched loci, only targets above 0.02 CPM in any condition for each chromatin modification were included as an input data.

*K*-means clustering was performed using base R, with cluster dimensionality visualized through PCA using the factoextra^80^ package. The optimal number of clusters was determined with the NbClust package^81^, reaching a consensus between three methods: Elbow, Silhouette and Gap statistics. To generate input data for *K*-means clustering, the GBP TAD and its neighboring regions, *KYAT3* and XR_947581 (region: chr1:88921060–89480060), were split into 10-kb bins. CPMs corresponding to each biological replicate, shared condition and histone mark from Cut&Run-seq were calculated for each bin and fed into the *K*-means clustering algorithm. The genomic coordinates of the bins for each cluster were mapped back to GBP loci and annotated manually.

#### Statistics

If not specified otherwise, the statistics for pairwise comparisons between conditions or samples were calculated using Student’s *t*-test in Microsoft Excel, GraphPad Prism or base R. Hetero- or homoscedasticity was determined using the *F*-test in Microsoft Excel or base R. The relevant significance levels are plotted as tabulated *P* values in each figure The error bars on bar plots throughout the paper are the s.e.m.

### Reporting summary

Further information on research design is available in the Nature Portfolio Reporting Summary linked to this article.

## Online content

Any methods, additional references, Nature Portfolio reporting summaries, source data, extended data, supplementary information, acknowledgements, peer review information; details of author contributions and competing interests; and statements of data and code availability are available at 10.1038/s41594-025-01522-8.

## Supplementary information

Reporting Summary

Supplementary Table Supplementary Table with primers and oligonucleotides.

## Source data

Source Data Fig. 1 Source data for plots and stats.

Source Data Fig. 2 Source data for plots and stats.

Source Data Fig. 3 Source data for plots and stats.

Source Data Fig. 4 Source data for plots and stats.

Source Data Fig. 5 Source data for plots and stats.

Source Data Fig. 6 Source data for plots and stats.

Source Data Fig. 7 Source data for plots and stats.

Source Data Extended Data Fig. 1 Source data for plots and stats.

Source Data Extended Data Fig. 1 Unprocessed western blots.

Source Data Extended Data Fig. 3 Source data for plots and stats.

Source Data Extended Data Fig. 4 Source data for plots and stats.

Source Data Extended Data Fig. 5 Source data for plots and stats.

Source Data Extended Data Fig. 8 Source data for plots and stats.

Source Data Extended Data Fig. 8 Unprocessed western blots.

Source Data Extended Data Fig. 9 Source data for plots and stats.

Source Data Extended Data Fig. 10 Source data for plots and stats.

## Data Availability

All sequencing data, raw reads and processed files, were deposited in Gene Expression Omnibus (GEO) and will be publicly accessible before publication at GEO Series record GSE249136. Source data are provided with this paper.

## Code availability

The scripts for the bioinformatic analyses with their parameters were deposited in public repository on GitHub under the link: https://github.com/Pwmski/mikulski-lab/tree/main/Mikulski-et-al-2023
