# Supplementary material for: Heritable maintenance of chromatin modifications confers transcriptional memory of interferon-γ signaling
Source: Nat Struct Mol Biol. 2025 Apr 4;32(7):1255–67. doi: 10.1038/s41594-025-01522-8 (PMC12263432; doi:10.1038/s41594-025-01522-8)
Supplement: Supplementary file 1 — Reporting Summary [file 41594_2025_1522_MOESM1_ESM.pdf]

nature portfolio

Corresponding author(s): Pawel Mikulski, Lars E.T. Jansen

Last updated by author(s): Jan 23, 2025

# Reporting Summary

Nature Portfolio wishes to improve the reproducibility of the work that we publish. This form provides structure for consistency and transparency in reporting. For further information on Nature Portfolio policies, see our Editorial Policies and the Editorial Policy Checklist.

## Statistics

For all statistical analyses, confirm that the following items are present in the figure legend, table legend, main text, or Methods section.

| n/a | Confirmed | |
|---|---|---|
| ☐ | ☒ | The exact sample size (*n*) for each experimental group/condition, given as a discrete number and unit of measurement |
| ☐ | ☒ | A statement on whether measurements were taken from distinct samples or whether the same sample was measured repeatedly |
| ☐ | ☒ | The statistical test(s) used AND whether they are one- or two-sided<br>*Only common tests should be described solely by name; describe more complex techniques in the Methods section.* |
| ☐ | ☒ | A description of all covariates tested |
| ☐ | ☒ | A description of any assumptions or corrections, such as tests of normality and adjustment for multiple comparisons |
| ☐ | ☒ | A full description of the statistical parameters including central tendency (e.g. means) or other basic estimates (e.g. regression coefficient) AND variation (e.g. standard deviation) or associated estimates of uncertainty (e.g. confidence intervals) |
| ☐ | ☒ | For null hypothesis testing, the test statistic (e.g. *F*, *t*, *r*) with confidence intervals, effect sizes, degrees of freedom and *P* value noted<br>*Give P values as exact values whenever suitable.* |
| ☒ | ☐ | For Bayesian analysis, information on the choice of priors and Markov chain Monte Carlo settings |
| ☒ | ☐ | For hierarchical and complex designs, identification of the appropriate level for tests and full reporting of outcomes |
| ☒ | ☐ | Estimates of effect sizes (e.g. Cohen's *d*, Pearson's *r*), indicating how they were calculated |

*Our web collection on statistics for biologists contains articles on many of the points above.*

## Software and code

Policy information about availability of computer code

| | |
|---|---|
| Data collection | The scripts for the bioinformatic analyses with their parameters were deposited in public repository on GitHub under the link: https://github.com/Pwmski/mikulski-lab/tree/main/Mikulski-et-al-2023. Data collection was performed with basespace-cli using scripts provided on aforementioned GitHub page. |
| Data analysis | The scripts for the bioinformatic analyses with their parameters were deposited in public repository on GitHub under the link: https://github.com/Pwmski/mikulski-lab/tree/main/Mikulski-et-al-2023. Data analysis was performed with unix-based tools: FastQC v0.11.9, Bowtie2, Samtools v1.1, Picard v3.1, Deeptools v2, Capcruncher pipeline and Cooler. Data analysis in R was performed with: factoextra v1.0.7, NbClust v3.0, VennDiagram v1.7.3. Post-counting data analysis and wrangling was performed with base R (R v4.1, RStudio v1.4) and R-based Tidyverse package collection (e.g. dplyr). Data visualization was performed with gglot2, ggrepel, IGV and WashU Epigenome Browser. FACS data was visualized and analyzed in FlowJo v10.8. |

For manuscripts utilizing custom algorithms or software that are central to the research but not yet described in published literature, software must be made available to editors and reviewers. We strongly encourage code deposition in a community repository (e.g. GitHub). See the Nature Portfolio guidelines for submitting code & software for further information.

# Data

Policy information about availability of data

All manuscripts must include a data availability statement. This statement should provide the following information, where applicable:

- Accession codes, unique identifiers, or web links for publicly available datasets
- A description of any restrictions on data availability
- For clinical datasets or third party data, please ensure that the statement adheres to our policy

All sequencing data, raw reads and processed files, were deposited in Gene Expression Omnibus (GEO) and are publicly accessible at GEO Series record GSE249136.

# Research involving human participants, their data, or biological material

Policy information about studies with human participants or human data. See also policy information about sex, gender (identity/presentation), and sexual orientation and race, ethnicity and racism.

| | |
|---|---|
| Reporting on sex and gender | n/a |
| Reporting on race, ethnicity, or other socially relevant groupings | n/a |
| Population characteristics | n/a |
| Recruitment | n/a |
| Ethics oversight | n/a |

Note that full information on the approval of the study protocol must also be provided in the manuscript.

# Field-specific reporting

Please select the one below that is the best fit for your research. If you are not sure, read the appropriate sections before making your selection.

☒ Life sciences ☐ Behavioural & social sciences ☐ Ecological, evolutionary & environmental sciences

For a reference copy of the document with all sections, see nature.com/documents/nr-reporting-summary-flat.pdf

# Life sciences study design

All studies must disclose on these points even when the disclosure is negative.

| | |
|---|---|
| Sample size | Sample size was set in alignment with standard guidelines, widely used in the field, e.g. Tehrani et al., EMBO J 2023. The sample size was set to n = 3 for RT-qPCR, Cut&Run-seq, FACS, 3C-qPCR and small molecule high-content microscopy. The samples size was set exceptionally to n = 2 in the experiments that were independently validated by alternative methodologies or further experiments and to preserve high sequencing coverage per sample in genomic experiments. These included: Capture-C-seq (validated by 3C-qPCR), selected Cut&Run-seq samples for H3K14ac and H3K27me3 (validated through at least two independent experiments with n=2 or 3 in each) and immunoblots (validated through at least two independent experiments). Each replicate corresponds to populational average from large number of cells (0.5 - 10 million, depending on the methodology). For experiments with single-cell output, the number of measured cells was >1000 (GBP1-GFP imaging) or >10000 (number of events in FACS). |
| Data exclusions | No data was excluded from the analysis. |
| Replication | Replication of results was ensured by validation by independent methodogies (e.g. FACS and microscopy for GBP1-GFP fluorescence measurements or 3C-qPCR and Capture-C), independent approaches (e.g. expression analyses of E2 in polyclonal and monoclonal mutant populations) and/or independent, repeated experiments (e.g. GBP1-GFP live-cell imaging in IFN induction timecourse). All experiments were replicated at least twice. The sample number and replicate number per each experiment were included in the figure legends. |
| Randomization | Randomization of samples was performed for all experiments. Randomization concerned cell growth (randomized arrangement in growth chambers and in tissue culture plates), cell collection (randomized order in processing samples) and sequencing library preparation (randomization in the usage of bardcode sets for multiplexing). |
| Blinding | Blinding was not relevant for the study. The study was not based on the prior assumptions on the responses to experiment treatments, nor involve clinical trials. For unbiased interpretation of data presented in the study, the results were interpreted and consulted independently by co-authors, collaborators and departmental bodies |

# Behavioural & social sciences study design

All studies must disclose on these points even when the disclosure is negative.

| | |
|---|---|
| Study description | *Briefly describe the study type including whether data are quantitative, qualitative, or mixed-methods (e.g. qualitative cross-sectional, quantitative experimental, mixed-methods case study).* |
| Research sample | *State the research sample (e.g. Harvard university undergraduates, villagers in rural India) and provide relevant demographic information (e.g. age, sex) and indicate whether the sample is representative. Provide a rationale for the study sample chosen. For studies involving existing datasets, please describe the dataset and source.* |
| Sampling strategy | *Describe the sampling procedure (e.g. random, snowball, stratified, convenience). Describe the statistical methods that were used to predetermine sample size OR if no sample-size calculation was performed, describe how sample sizes were chosen and provide a rationale for why these sample sizes are sufficient. For qualitative data, please indicate whether data saturation was considered, and what criteria were used to decide that no further sampling was needed.* |
| Data collection | *Provide details about the data collection procedure, including the instruments or devices used to record the data (e.g. pen and paper, computer, eye tracker, video or audio equipment) whether anyone was present besides the participant(s) and the researcher, and whether the researcher was blind to experimental condition and/or the study hypothesis during data collection.* |
| Timing | *Indicate the start and stop dates of data collection. If there is a gap between collection periods, state the dates for each sample cohort.* |
| Data exclusions | *If no data were excluded from the analyses, state so OR if data were excluded, provide the exact number of exclusions and the rationale behind them, indicating whether exclusion criteria were pre-established.* |
| Non-participation | *State how many participants dropped out/declined participation and the reason(s) given OR provide response rate OR state that no participants dropped out/declined participation.* |
| Randomization | *If participants were not allocated into experimental groups, state so OR describe how participants were allocated to groups, and if allocation was not random, describe how covariates were controlled.* |

# Ecological, evolutionary & environmental sciences study design

All studies must disclose on these points even when the disclosure is negative.

| | |
|---|---|
| Study description | *Briefly describe the study. For quantitative data include treatment factors and interactions, design structure (e.g. factorial, nested, hierarchical), nature and number of experimental units and replicates.* |
| Research sample | *Describe the research sample (e.g. a group of tagged Passer domesticus, all Stenocereus thurberi within Organ Pipe Cactus National Monument), and provide a rationale for the sample choice. When relevant, describe the organism taxa, source, sex, age range and any manipulations. State what population the sample is meant to represent when applicable. For studies involving existing datasets, describe the data and its source.* |
| Sampling strategy | *Note the sampling procedure. Describe the statistical methods that were used to predetermine sample size OR if no sample-size calculation was performed, describe how sample sizes were chosen and provide a rationale for why these sample sizes are sufficient.* |
| Data collection | *Describe the data collection procedure, including who recorded the data and how.* |
| Timing and spatial scale | *Indicate the start and stop dates of data collection, noting the frequency and periodicity of sampling and providing a rationale for these choices. If there is a gap between collection periods, state the dates for each sample cohort. Specify the spatial scale from which the data are taken* |
| Data exclusions | *If no data were excluded from the analyses, state so OR if data were excluded, describe the exclusions and the rationale behind them, indicating whether exclusion criteria were pre-established.* |
| Reproducibility | *Describe the measures taken to verify the reproducibility of experimental findings. For each experiment, note whether any attempts to repeat the experiment failed OR state that all attempts to repeat the experiment were successful.* |
| Randomization | *Describe how samples/organisms/participants were allocated into groups. If allocation was not random, describe how covariates were controlled. If this is not relevant to your study, explain why.* |
| Blinding | *Describe the extent of blinding used during data acquisition and analysis. If blinding was not possible, describe why OR explain why blinding was not relevant to your study.* |

Did the study involve field work? ☐ Yes ☐ No

## Field work, collection and transport

| | |
|---|---|
| Field conditions | *Describe the study conditions for field work, providing relevant parameters (e.g. temperature, rainfall).* |
| Location | *State the location of the sampling or experiment, providing relevant parameters (e.g. latitude and longitude, elevation, water depth).* |
| Access & import/export | *Describe the efforts you have made to access habitats and to collect and import/export your samples in a responsible manner and in compliance with local, national and international laws, noting any permits that were obtained (give the name of the issuing authority, the date of issue, and any identifying information).* |
| Disturbance | *Describe any disturbance caused by the study and how it was minimized.* |

# Reporting for specific materials, systems and methods

We require information from authors about some types of materials, experimental systems and methods used in many studies. Here, indicate whether each material, system or method listed is relevant to your study. If you are not sure if a list item applies to your research, read the appropriate section before selecting a response.

### Materials & experimental systems

| n/a | Involved in the study | |
|---|---|---|
| ☐ | ☒ | Antibodies |
| ☐ | ☒ | Eukaryotic cell lines |
| ☒ | ☐ | Palaeontology and archaeology |
| ☒ | ☐ | Animals and other organisms |
| ☒ | ☐ | Clinical data |
| ☒ | ☐ | Dual use research of concern |
| ☒ | ☐ | Plants |

### Methods

| n/a | Involved in the study | |
|---|---|---|
| ☐ | ☒ | ChIP-seq |
| ☐ | ☒ | Flow cytometry |
| ☒ | ☐ | MRI-based neuroimaging |

## Antibodies

Antibodies used

The antibodies used were: -H3K4me1 (Epicypher, #13-0057), -H3K4me3 (Epicypher, #13-0041), -H3K14ac (Merck, #07-353), -H4K16ac (Merck, #07-329), -H3K27me3 (Cell Signaling Technology, #9733), -Kat7 (Abcam, #ab190908); -alpha tubulin (Sigma, #T9026), -H3 (Abcam, #ab1791). The antibodies were used at dilutions according to the manufacturers' manuals, e.g. 0.5 ug per IP for Cut&Run.

Validation

H3Kme1 and H3K4me3 antibodies were validated by a provider (Epicypher) with the following statements:
This H3K4me1 (histone H3 lysine 4 monomethyl) antibody meets EpiCypher's lot-specific SNAP-Certified™ criteria for specificity and efficient target enrichment in both CUT&RUN and CUT&Tag applications. This requires <20% cross-reactivity to related histone PTMs determined using the SNAP-CUTANA™ K-MetStat Panel of spike-in controls (EpiCypher 19-1002). High target efficiency is confirmed by consistent genomic enrichment at varying cell inputs: 500k and 50k cells in CUT&RUN; 100k and 10k cells in CUT&Tag. High efficiency antibodies display similar peak structures at representative loci and highly conserved genome-wide signal even at reduced cell numbers. H3K4me1 either flanks H3K4me3 at the transcription start site (TSS) or coincides with H3K4me3.
This H3K4me3 (histone H3 lysine 4 trimethyl) antibody meets EpiCypher's lot-specific SNAP-Certified™ criteria for specificity and efficient target enrichment in CUT&RUN. This requires <20% cross-reactivity to related histone PTMs determined using the SNAP-CUTANA™ K-MetStat Panel of spike-in controls (EpiCypher 19-1002). High target efficiency is confirmed by consistent genomic enrichment at 500k and 50k starting cells. This antibody targets histone H3 trimethylated at lysine 4, which is enriched at active promoters near transcription start sites (TSS).
H3K14ac and H4K16ac antibodies were validated by a provider (Merck) with the following statements:
H3K14ac – quality level MQ100. Quality assurance - in acid extracts from sodium butyrate treated HeLa cells. A 1:1000-1:5000 dilution of this lot detected acetyl-Histone H3 (Lys14) in acid extracts from sodium butyrate treated HeLa cells (Catalog # 17-305) in Western Blot.
H4K16ac – quality level MQ100. Quality assurance - evaluated by Western Blotting in lysate from Sodium Butyrate treated HeLa cells. A 1:1,000 dilution of this antibody detected Acetyl-Histone H4 (Lys16) in lysates from Sodium Butyrate treated HeLa cells in Western Blot.
H3K27ac antibody were validated by a provider (CST) with the following statement:
Tri-Methyl-Histone H3 (Lys27) (C36B11) Rabbit mAb detects endogenous levels of histone H3 only when tri-methylated on Lys27. The antibody does not cross-react with non-methylated, mono-methylated or di-methylated Lys27. In addition, the antibody does not cross-react with mono-methylated, di-methylated or tri-methylated histone H3 at Lys4, Lys9, Lys36 or Histone H4 at Lys20. This antibody has been validated using SimpleChIP® Enzymatic Chromatin IP Kits.
Kat7 antibody were validated by a provider (Abcam) with the following statement:
Antibody tested for human reactivity and validated by IP, WB, ICC/IF and IHC-P. This product is a recombinant monoclonal antibody derived from reproducible hybridoma technology.

Alpha-tubulin antibody were validated by a provider (Sigma) with the following statement:
The product is Enhanced Validation Antibody.

H3 antibody were validated by a provider (Abcam) with the following statement:
Antibody tested for human reactivity and validated by WB, ChIP, ICC/IF. The product has expected reactivity by IHC-P and IP.

H3Kme1 and H3K4me3 antibodies were validated by a provider (Epicypher) with the following statements:
This H3K4me1 (histone H3 lysine 4 monomethyl) antibody meets EpiCypher's lot-specific SNAP-Certified™ criteria for specificity and efficient target enrichment in both CUT&RUN and CUT&Tag applications. This requires <20% cross-reactivity to related histone PTMs determined using the SNAP-CUTANA™ K-MetStat Panel of spike-in controls (EpiCypher 19-1002). High target efficiency is confirmed by consistent genomic enrichment at varying cell inputs: 500k and 50k cells in CUT&RUN; 100k and 10k cells in CUT&Tag. High efficiency antibodies display similar peak structures at representative loci and highly conserved genome-wide signal even at reduced cell numbers. H3K4me1 either flanks H3K4me3 at the transcription start site (TSS) or coincides with H3K4me3.
This H3K4me3 (histone H3 lysine 4 trimethyl) antibody meets EpiCypher's lot-specific SNAP-Certified™ criteria for specificity and efficient target enrichment in CUT&RUN. This requires <20% cross-reactivity to related histone PTMs determined using the SNAP-CUTANA™ K-MetStat Panel of spike-in controls (EpiCypher 19-1002). High target efficiency is confirmed by consistent genomic enrichment at 500k and 50k starting cells. This antibody targets histone H3 trimethylated at lysine 4, which is enriched at active promoters near transcription start sites (TSS).
H3K14ac and H4K16ac antibodies were validated by a provider (Merck) with the following statements:
H3K14ac – quality level MQ100. Quality assurance - in acid extracts from sodium butyrate treated HeLa cells. A 1:1000-1:5000 dilution of this lot detected acetyl-Histone H3 (Lys14) in acid extracts from sodium butyrate treated HeLa cells (Catalog # 17-305) in Western Blot.
H4K16ac – quality level MQ100. Quality assurance - evaluated by Western Blotting in lysate from Sodium Butyrate treated HeLa cells. A 1:1,000 dilution of this antibody detected Acetyl-Histone H4 (Lys16) in lysates from Sodium Butyrate treated HeLa cells in Western Blot.
H3K27ac antibody were validated by a provider (CST) with the following statement:
Tri-Methyl-Histone H3 (Lys27) (C36B11) Rabbit mAb detects endogenous levels of histone H3 only when tri-methylated on Lys27. The antibody does not cross-react with non-methylated, mono-methylated or di-methylated Lys27. In addition, the antibody does not cross-react with mono-methylated, di-methylated or tri-methylated histone H3 at Lys4, Lys9, Lys36 or Histone H4 at Lys20. This antibody has been validated using SimpleChIP® Enzymatic Chromatin IP Kits.
Kat7 antibody were validated by a provider (Abcam) with the following statement:
Antibody tested for human reactivity and validated by IP, WB, ICC/IF and IHC-P. This product is a recombinant monoclonal antibody derived from reproducible hybridoma technology.

Alpha-tubulin antibody were validated by a provider (Sigma) with the following statement:
The product is Enhanced Validation Antibody.
H3 antibody were validated by a provider (Abcam) with the following statement:
Antibody tested for human reactivity and validated by WB, ChIP, ICC/IF. The product has expected reactivity by IHC-P and IP.

H3Kme1 and H3K4me3 antibodies were validated by a provider (Epicypher) with the following statements:
This H3K4me1 (histone H3 lysine 4 monomethyl) antibody meets EpiCypher's lot-specific SNAP-Certified™ criteria for specificity and efficient target enrichment in both CUT&RUN and CUT&Tag applications. This requires <20% cross-reactivity to related histone PTMs determined using the SNAP-CUTANA™ K-MetStat Panel of spike-in controls (EpiCypher 19-1002). High target efficiency is confirmed by consistent genomic enrichment at varying cell inputs: 500k and 50k cells in CUT&RUN; 100k and 10k cells in CUT&Tag. High efficiency antibodies display similar peak structures at representative loci and highly conserved genome-wide signal even at reduced cell numbers. H3K4me1 either flanks H3K4me3 at the transcription start site (TSS) or coincides with H3K4me3.
This H3K4me3 (histone H3 lysine 4 trimethyl) antibody meets EpiCypher's lot-specific SNAP-Certified™ criteria for specificity and efficient target enrichment in CUT&RUN. This requires <20% cross-reactivity to related histone PTMs determined using the SNAP-CUTANA™ K-MetStat Panel of spike-in controls (EpiCypher 19-1002). High target efficiency is confirmed by consistent genomic enrichment at 500k and 50k starting cells. This antibody targets histone H3 trimethylated at lysine 4, which is enriched at active promoters near transcription start sites (TSS).
H3K14ac and H4K16ac antibodies were validated by a provider (Merck) with the following statements:
H3K14ac – quality level MQ100. Quality assurance - in acid extracts from sodium butyrate treated HeLa cells. A 1:1000-1:5000 dilution of this lot detected acetyl-Histone H3 (Lys14) in acid extracts from sodium butyrate treated HeLa cells (Catalog # 17-305) in Western Blot.
H4K16ac – quality level MQ100. Quality assurance - evaluated by Western Blotting in lysate from Sodium Butyrate treated HeLa cells. A 1:1,000 dilution of this antibody detected Acetyl-Histone H4 (Lys16) in lysates from Sodium Butyrate treated HeLa cells in Western Blot.
H3K27ac antibody were validated by a provider (CST) with the following statement:
Tri-Methyl-Histone H3 (Lys27) (C36B11) Rabbit mAb detects endogenous levels of histone H3 only when tri-methylated on Lys27. The antibody does not cross-react with non-methylated, mono-methylated or di-methylated Lys27. In addition, the antibody does not cross-react with mono-methylated, di-methylated or tri-methylated histone H3 at Lys4, Lys9, Lys36 or Histone H4 at Lys20. This antibody has been validated using SimpleChIP® Enzymatic Chromatin IP Kits.
Kat7 antibody were validated by a provider (Abcam) with the following statement:
Antibody tested for human reactivity and validated by IP, WB, ICC/IF and IHC-P. This product is a recombinant monoclonal antibody derived from reproducible hybridoma technology.

Alpha-tubulin antibody were validated by a provider (Sigma) with the following statement:
The product is Enhanced Validation Antibody.
H3 antibody were validated by a provider (Abcam) with the following statement:
H3Kme1 and H3K4me3 antibodies were validated by a provider (Epicypher) with the following statements:
This H3K4me1 (histone H3 lysine 4 monomethyl) antibody meets EpiCypher's lot-specific SNAP-Certified™ criteria for specificity and efficient target enrichment in both CUT&RUN and CUT&Tag applications. This requires <20% cross-reactivity to related histone PTMs determined using the SNAP-CUTANA™ K-MetStat Panel of spike-in controls (EpiCypher 19-1002). High target efficiency is confirmed by consistent genomic enrichment at varying cell inputs: 500k and 50k cells in CUT&RUN; 100k and 10k cells in CUT&Tag. High

efficiency antibodies display similar peak structures at representative loci and highly conserved genome-wide signal even at reduced cell numbers. H3K4me1 either flanks H3K4me3 at the transcription start site (TSS) or coincides with H3K4me3.
This H3K4me3 (histone H3 lysine 4 trimethyl) antibody meets EpiCypher's lot-specific SNAP-Certified™ criteria for specificity and efficient target enrichment in CUT&RUN. This requires <20% cross-reactivity to related histone PTMs determined using the SNAP-CUTANA™ K-MetStat Panel of spike-in controls (EpiCypher 19-1002). High target efficiency is confirmed by consistent genomic enrichment at 500k and 50k starting cells. This antibody targets histone H3 trimethylated at lysine 4, which is enriched at active promoters near transcription start sites (TSS).
H3K14ac and H4K16ac antibodies were validated by a provider (Merck) with the following statements:
H3K14ac – quality level MQ100. Quality assurance - in acid extracts from sodium butyrate treated HeLa cells. A 1:1000-1:5000 dilution of this lot detected acetyl-Histone H3 (Lys14) in acid extracts from sodium butyrate treated HeLa cells (Catalog # 17-305) in Western Blot.
H4K16ac – quality level MQ100. Quality assurance - evaluated by Western Blotting in lysate from Sodium Butyrate treated HeLa cells. A 1:1,000 dilution of this antibody detected Acetyl-Histone H4 (Lys16) in lysates from Sodium Butyrate treated HeLa cells in Western Blot.
H3K27ac antibody were validated by a provider (CST) with the following statement:
Tri-Methyl-Histone H3 (Lys27) (C36B11) Rabbit mAb detects endogenous levels of histone H3 only when tri-methylated on Lys27. The antibody does not cross-react with non-methylated, mono-methylated or di-methylated Lys27. In addition, the antibody does not cross-react with mono-methylated, di-methylated or tri-methylated histone H3 at Lys4, Lys9, Lys36 or Histone H4 at Lys20. This antibody has been validated using SimpleChIP® Enzymatic Chromatin IP Kits.
Kat7 antibody were validated by a provider (Abcam) with the following statement:
Antibody tested for human reactivity and validated by IP, WB, ICC/IF and IHC-P. This product is a recombinant monoclonal antibody derived from reproducible hybridoma technology.

Alpha-tubulin antibody were validated by a provider (Sigma) with the following statement:
The product is Enhanced Validation Antibody.
H3 antibody were validated by a provider (Abcam) with the following statement:
Antibody tested for human reactivity and validated by WB, ChIP, ICC/IF. The product has expected reactivity by IHC-P and IP.

## Eukaryotic cell lines

Policy information about cell lines and Sex and Gender in Research

Cell line source(s): HeLa Kyoto line (female, RRID: CVCL_1922), obtained from EMBL; THP-1 (RRID:CVCL_0006) obtained from ATCC (TIB-202); Human primary neonatal fibroblast obtained from Lonza CC-2509

Authentication: HeLa cell transgenic lines were PCR and Western authenticated. Commercial cell lines authenticated at source

Mycoplasma contamination: The cell line used in the study has been routinely tested for mycoplasma contamination. The cell line used in the study tested negative.

Commonly misidentified lines (See ICLAC register): The line used in this study is not a misidentified line, according to ICLAC.

## Palaeontology and Archaeology

Specimen provenance: *Provide provenance information for specimens and describe permits that were obtained for the work (including the name of the issuing authority, the date of issue, and any identifying information). Permits should encompass collection and, where applicable, export.*

Specimen deposition: *Indicate where the specimens have been deposited to permit free access by other researchers.*

Dating methods: *If new dates are provided, describe how they were obtained (e.g. collection, storage, sample pretreatment and measurement), where they were obtained (i.e. lab name), the calibration program and the protocol for quality assurance OR state that no new dates are provided.*

☐ Tick this box to confirm that the raw and calibrated dates are available in the paper or in Supplementary Information.

Ethics oversight: *Identify the organization(s) that approved or provided guidance on the study protocol, OR state that no ethical approval or guidance was required and explain why not.*

Note that full information on the approval of the study protocol must also be provided in the manuscript.

## Animals and other research organisms

Policy information about studies involving animals; ARRIVE guidelines recommended for reporting animal research, and Sex and Gender in Research

Laboratory animals: *For laboratory animals, report species, strain and age OR state that the study did not involve laboratory animals.*

Wild animals: *Provide details on animals observed in or captured in the field; report species and age where possible. Describe how animals were*

| | |
|---|---|
| Wild animals | *caught and transported and what happened to captive animals after the study (if killed, explain why and describe method; if released, say where and when) OR state that the study did not involve wild animals.* |
| Reporting on sex | *Indicate if findings apply to only one sex; describe whether sex was considered in study design, methods used for assigning sex. Provide data disaggregated for sex where this information has been collected in the source data as appropriate; provide overall numbers in this Reporting Summary. Please state if this information has not been collected. Report sex-based analyses where performed, justify reasons for lack of sex-based analysis.* |
| Field-collected samples | *For laboratory work with field-collected samples, describe all relevant parameters such as housing, maintenance, temperature, photoperiod and end-of-experiment protocol OR state that the study did not involve samples collected from the field.* |
| Ethics oversight | *Identify the organization(s) that approved or provided guidance on the study protocol, OR state that no ethical approval or guidance was required and explain why not.* |

Note that full information on the approval of the study protocol must also be provided in the manuscript.

# Clinical data

Policy information about clinical studies

All manuscripts should comply with the ICMJE guidelines for publication of clinical research and a completed CONSORT checklist must be included with all submissions.

| | |
|---|---|
| Clinical trial registration | *Provide the trial registration number from ClinicalTrials.gov or an equivalent agency.* |
| Study protocol | *Note where the full trial protocol can be accessed OR if not available, explain why.* |
| Data collection | *Describe the settings and locales of data collection, noting the time periods of recruitment and data collection.* |
| Outcomes | *Describe how you pre-defined primary and secondary outcome measures and how you assessed these measures.* |

# Dual use research of concern

Policy information about dual use research of concern

## Hazards

Could the accidental, deliberate or reckless misuse of agents or technologies generated in the work, or the application of information presented in the manuscript, pose a threat to:

| No | Yes | |
|---|---|---|
| ☒ | ☐ | Public health |
| ☒ | ☐ | National security |
| ☒ | ☐ | Crops and/or livestock |
| ☒ | ☐ | Ecosystems |
| ☒ | ☐ | Any other significant area |

## Experiments of concern

Does the work involve any of these experiments of concern:

| No | Yes | |
|---|---|---|
| ☒ | ☐ | Demonstrate how to render a vaccine ineffective |
| ☒ | ☐ | Confer resistance to therapeutically useful antibiotics or antiviral agents |
| ☒ | ☐ | Enhance the virulence of a pathogen or render a nonpathogen virulent |
| ☒ | ☐ | Increase transmissibility of a pathogen |
| ☒ | ☐ | Alter the host range of a pathogen |
| ☒ | ☐ | Enable evasion of diagnostic/detection modalities |
| ☒ | ☐ | Enable the weaponization of a biological agent or toxin |
| ☒ | ☐ | Any other potentially harmful combination of experiments and agents |

# Plants

Seed stocks

n/a

Novel plant genotypes

n/a

Authentication

n/a

# ChIP-seq

## Data deposition

☒ Confirm that both raw and final processed data have been deposited in a public database such as GEO.

☐ Confirm that you have deposited or provided access to graph files (e.g. BED files) for the called peaks.

Data access links
*May remain private before publication.*

Cut&Run-seq was performed instead of related ChIP-seq method. All sequencing data, raw reads and processed files, were deposited in Gene Expression Omnibus (GEO) at GEO Series record GSE249136.

Files in database submission

s1_rmvdups_k4me3_c1-1.bw; s1_k4me3_r1.fastq.gz; s1_k4me3_r2.fastq.gz
s2_rmvdups_k4me3_c1-2.bw; s2_k4me3_r1.fastq.gz; s2_k4me3_r2.fastq.gz
s3_rmvdups_k4me3_c1-3.bw; s3_k4me3_r1.fastq.gz; s3_k4me3_r2.fastq.gz
s8_rmvdups_k4me3_c3-2.bw; s8_k4me3_r1.fastq.gz; s8_k4me3_r2.fastq.gz
s9_rmvdups_k4me3_c3-3.bw; s9_k4me3_r1.fastq.gz; s9_k4me3_r2.fastq.gz
s4_rmvdups_k4me3_c5-1.bw; s4_k4me3_r1.fastq.gz; s4_k4me3_r2.fastq.gz
s5_rmvdups_k4me3_c5-2.bw; s5_k4me3_r1.fastq.gz; s5_k4me3_r2.fastq.gz
s13_rmvdups_k4me3_c55-1.bw; s13_k4me3_r1.fastq.gz; s13_k4me3_r2.fastq.gz
s14_rmvdups_k4me3_c55-2.bw; s14_k4me3_r1.fastq.gz; s14_k4me3_r2.fastq.gz
s15_rmvdups_k4me3_c55-3.bw; s15_k4me3_r1.fastq.gz; s15_k4me3_r2.fastq.gz
s10_rmvdups_k4me3_c7-1.bw; s10_k4me3_r1.fastq.gz; s10_k4me3_r2.fastq.gz
s11_rmvdups_k4me3_c7-2.bw; s11_k4me3_r1.fastq.gz; s11_k4me3_r2.fastq.gz
s1_rmvdups_K4me1_c1-1.bw; s1_k4me1_r1.fastq.gz; s1_k4me1_r2.fastq.gz
s2_rmvdups_K4me1_c1-2.bw; s2_k4me1_r1.fastq.gz; s2_k4me1_r2.fastq.gz
s3_rmvdups_K4me1_c1-3.bw; s3_k4me1_r1.fastq.gz; s3_k4me1_r2.fastq.gz
s7_rmvdups_K4me1_c3-1.bw; s7_k4me1_r1.fastq.gz; s7_k4me1_r2.fastq.gz
s8_rmvdups_K4me1_c3-2.bw; s8_k4me1_r1.fastq.gz; s8_k4me1_r2.fastq.gz
s9_rmvdups_K4me1_c3-3.bw; s9_k4me1_r1.fastq.gz; s9_k4me1_r2.fastq.gz
s4_rmvdups_K4me1_c5-1.bw; s4_k4me1_r1.fastq.gz; s4_k4me1_r2.fastq.gz
s5_rmvdups_K4me1_c5-2.bw; s5_k4me1_r1.fastq.gz; s5_k4me1_r2.fastq.gz
s6_rmvdups_K4me1_c5-3.bw; s6_k4me1_r1.fastq.gz; s6_k4me1_r2.fastq.gz
s14_rmvdups_K4me1_c55-2.bw; s14_k4me1_r1.fastq.gz; s14_k4me1_r2.fastq.gz
s15_rmvdups_K4me1_c55-3.bw; s15_k4me1_r1.fastq.gz; s15_k4me1_r2.fastq.gz
s10_rmvdups_K4me1_c7-1.bw; s10_k4me1_r1.fastq.gz; s10_k4me1_r2.fastq.gz
s11_rmvdups_K4me1_c7-2.bw; s11_k4me1_r1.fastq.gz; s11_k4me1_r2.fastq.gz
s1_rmvdups_h3k14ac_c1-1.bw; s1_h3k14ac_r1.fastq.gz; s1_h3k14ac_r2.fastq.gz
s2_rmvdups_h3k14ac_c1-2.bw; s2_h3k14ac_r1.fastq.gz; s2_h3k14ac_r2.fastq.gz
s3_rmvdups_h3k14ac_c1-3.bw; s3_h3k14ac_r1.fastq.gz; s3_h3k14ac_r2.fastq.gz
s7_rmvdups_h3k14ac_c3-1.bw; s7_h3k14ac_r1.fastq.gz; s7_h3k14ac_r2.fastq.gz
s8_rmvdups_h3k14ac_c3-2.bw; s8_h3k14ac_r1.fastq.gz; s8_h3k14ac_r2.fastq.gz
s9_rmvdups_h3k14ac_c3-3.bw; s9_h3k14ac_r1.fastq.gz; s9_h3k14ac_r2.fastq.gz
s4_rmvdups_h3k14ac_c5-1.bw; s4_h3k14ac_r1.fastq.gz; s4_h3k14ac_r2.fastq.gz
s5_rmvdups_h3k14ac_c5-2.bw; s5_h3k14ac_r1.fastq.gz; s5_h3k14ac_r2.fastq.gz
s6_rmvdups_h3k14ac_c5-3.bw; s6_h3k14ac_r1.fastq.gz; s6_h3k14ac_r2.fastq.gz
s13_rmvdups_h3k14ac_c55-1.bw; s13_h3k14ac_r1.fastq.gz; s13_h3k14ac_r2.fastq.gz
s14_rmvdups_h3k14ac_c55-2.bw; s14_h3k14ac_r1.fastq.gz; s14_h3k14ac_r2.fastq.gz
s15_rmvdups_h3k14ac_c55-3.bw; s15_h3k14ac_r1.fastq.gz; s15_h3k14ac_r2.fastq.gz
s10_rmvdups_h3k14ac_c7-1.bw; s10_h3k14ac_r1.fastq.gz; s10_h3k14ac_r2.fastq.gz
s11_rmvdups_h3k14ac_c7-2.bw; s11_h3k14ac_r1.fastq.gz; s11_h3k14ac_r2.fastq.gz
s12_rmvdups_h3k14ac_c7-3.bw; s12_h3k14ac_r1.fastq.gz; s12_h3k14ac_r2.fastq.gz
s17_rmvdups_h4k16ac_c1-1.bw; s17_h4k16ac_r1.fastq.gz; s17_h4k16ac_r2.fastq.gz
s18_rmvdups_h4k16ac_c1-2.bw; s18_h4k16ac_r1.fastq.gz; s18_h4k16ac_r2.fastq.gz
s19_rmvdups_h4k16ac_c1-3.bw; s19_h4k16ac_r1.fastq.gz; s19_h4k16ac_r2.fastq.gz
s23_rmvdups_h4k16ac_c3-1.bw; s23_h4k16ac_r1.fastq.gz; s23_h4k16ac_r2.fastq.gz
s24_rmvdups_h4k16ac_c3-2.bw; s24_h4k16ac_r1.fastq.gz; s24_h4k16ac_r2.fastq.gz
s25_rmvdups_h4k16ac_c3-3.bw; s25_h4k16ac_r1.fastq.gz; s25_h4k16ac_r2.fastq.gz
s20_rmvdups_h4k16ac_c5-1.bw; s20_h4k16ac_r1.fastq.gz; s20_h4k16ac_r2.fastq.gz

s21_rmvdups_h4k16ac_c5-2.bw; s21_h4k16ac_r1.fastq.gz; s21_h4k16ac_r2.fastq.gz
s22_rmvdups_h4k16ac_c5-3.bw; s22_h4k16ac_r1.fastq.gz; s22_h4k16ac_r2.fastq.gz
s29_rmvdups_h4k16ac_c55-1.bw; s29_h4k16ac_r1.fastq.gz; s29_h4k16ac_r2.fastq.gz
s30_rmvdups_h4k16ac_c55-2.bw; s30_h4k16ac_r1.fastq.gz; s30_h4k16ac_r2.fastq.gz
s31_rmvdups_h4k16ac_c55-3.bw; s31_h4k16ac_r1.fastq.gz; s31_h4k16ac_r2.fastq.gz
s26_rmvdups_h4k16ac_c7-1.bw; s26_h4k16ac_r1.fastq.gz; s26_h4k16ac_r2.fastq.gz
s27_rmvdups_h4k16ac_c7-2.bw; s27_h4k16ac_r1.fastq.gz; s27_h4k16ac_r2.fastq.gz
s28_rmvdups_h4k16ac_c7-3.bw; s28_h4k16ac_r1.fastq.gz; s28_h4k16ac_r2.fastq.gz
s1_h3k27me3_timecourse_rmvdups.bw; s1_h3k27me3_timecourse_r1.fastq.gz; s1_h3k27me3_timecourse_r2.fastq.gz
s2_h3k27me3_timecourse_rmvdups.bw; s2_h3k27me3_timecourse_r1.fastq.gz; s2_h3k27me3_timecourse_r2.fastq.gz
s3_h3k27me3_timecourse_rmvdups.bw; s3_h3k27me3_timecourse_r1.fastq.gz; s3_h3k27me3_timecourse_r2.fastq.gz
s4_h3k27me3_timecourse_rmvdups.bw; s4_h3k27me3_timecourse_r1.fastq.gz; s4_h3k27me3_timecourse_r2.fastq.gz
s5_h3k27me3_timecourse_rmvdups.bw; s5_h3k27me3_timecourse_r1.fastq.gz; s5_h3k27me3_timecourse_r2.fastq.gz
s6_h3k27me3_timecourse_rmvdups.bw; s6_h3k27me3_timecourse_r1.fastq.gz; s6_h3k27me3_timecourse_r2.fastq.gz
s7_h3k27me3_timecourse_rmvdups.bw; s7_h3k27me3_timecourse_r1.fastq.gz; s7_h3k27me3_timecourse_r2.fastq.gz
s9_h3k27me3_timecourse_rmvdups.bw; s9_h3k27me3_timecourse_r1.fastq.gz; s9_h3k27me3_timecourse_r2.fastq.gz
s10_h3k27me3_timecourse_rmvdups.bw; s10_h3k27me3_timecourse_r1.fastq.gz; s10_h3k27me3_timecourse_r2.fastq.gz
s11_h3k27me3_timecourse_rmvdups.bw; s11_h3k27me3_timecourse_r1.fastq.gz; s11_h3k27me3_timecourse_r2.fastq.gz
s12_h3k27me3_timecourse_rmvdups.bw; s12_h3k27me3_timecourse_r1.fastq.gz; s12_h3k27me3_timecourse_r2.fastq.gz
s13_h3k27me3_timecourse_rmvdups.bw; s13_h3k27me3_timecourse_r1.fastq.gz; s13_h3k27me3_timecourse_r2.fastq.gz
s14_h3k27me3_timecourse_rmvdups.bw; s14_h3k27me3_timecourse_r1.fastq.gz; s14_h3k27me3_timecourse_r2.fastq.gz
s1_h3k14ac_timecourse_rmvdups.bw; s1_h3k14ac_timecourse_r1.fastq.gz; s1_h3k14ac_timecourse_r2.fastq.gz
s2_h3k14ac_timecourse_rmvdups.bw; s2_h3k14ac_timecourse_r1.fastq.gz; s2_h3k14ac_timecourse_r2.fastq.gz
s3_h3k14ac_timecourse_rmvdups.bw; s3_h3k14ac_timecourse_r1.fastq.gz; s3_h3k14ac_timecourse_r2.fastq.gz
s4_h3k14ac_timecourse_rmvdups.bw; s4_h3k14ac_timecourse_r1.fastq.gz; s4_h3k14ac_timecourse_r2.fastq.gz
s5_h3k14ac_timecourse_rmvdups.bw; s5_h3k14ac_timecourse_r1.fastq.gz; s5_h3k14ac_timecourse_r2.fastq.gz
s6_h3k14ac_timecourse_rmvdups.bw; s6_h3k14ac_timecourse_r1.fastq.gz; s6_h3k14ac_timecourse_r2.fastq.gz
s7_h3k14ac_timecourse_rmvdups.bw; s7_h3k14ac_timecourse_r1.fastq.gz; s7_h3k14ac_timecourse_r2.fastq.gz
s8_h3k14ac_timecourse_rmvdups.bw; s8_h3k14ac_timecourse_r1.fastq.gz; s8_h3k14ac_timecourse_r2.fastq.gz
s9_h3k14ac_timecourse_rmvdups.bw; s9_h3k14ac_timecourse_r1.fastq.gz; s9_h3k14ac_timecourse_r2.fastq.gz
s10_h3k14ac_timecourse_rmvdups.bw; s10_h3k14ac_timecourse_r1.fastq.gz; s10_h3k14ac_timecourse_r2.fastq.gz
s11_h3k14ac_timecourse_rmvdups.bw; s11_h3k14ac_timecourse_r1.fastq.gz; s11_h3k14ac_timecourse_r2.fastq.gz
s12_h3k14ac_timecourse_rmvdups.bw; s12_h3k14ac_timecourse_r1.fastq.gz; s12_h3k14ac_timecourse_r2.fastq.gz
s13_h3k14ac_timecourse_rmvdups.bw; s13_h3k14ac_timecourse_r1.fastq.gz; s13_h3k14ac_timecourse_r2.fastq.gz
s14_h3k14ac_timecourse_rmvdups.bw; s14_h3k14ac_timecourse_r1.fastq.gz; s14_h3k14ac_timecourse_r2.fastq.gz

Genome browser session (e.g. UCSC): n/a (submission record contains bigwig files for easy visualization in IGV or WashU Epigenome Browser)

### Methodology

**Replicates**: The study uses individual biological replicates (n = 2 or 3, depending on the experiment). A replicate is a sample harvested from separate cell culture plate.

**Sequencing depth**: Sequencing was performed Illumina NextSeq 500 as pair-end short-read sequencing with 75bp read length (2x 75bp) and Mid-Output (Cut&Run). The sequencing depth per sample across all experiments and samples was within 13.0 - 30.6 million reads.

**Antibodies**: The antibodies used were: α-H3K4me1 (Epicypher, #13-0057), α-H3K4me3 (Epicypher, #13-0041), α-H3K14ac (Merck, #07-353), α-H4K16ac (Merck, #07-329), α-H3K27me3 (Cell Signaling Technology, #9733).

**Peak calling parameters**: Peak calling was not performed. More relevant for the study was read counting for specified genomic regions. Read counting and cross-comparison between samples was performed with Deeptools v2 (multibigwig summary and bamcoverage commands). The parameters of all tools were included on GitHub page specified in Code Availability Statement.

**Data quality**: Data quality was assessed using Basespace and FastQC.

**Software**: Cut&Run-seq was analyzed using FastQC, Bowtie2, Samtools v1.1, Picard, Deeptools v2 and Tidyverse package collection.

## Flow Cytometry

### Plots

Confirm that:

☒ The axis labels state the marker and fluorochrome used (e.g. CD4-FITC).

☒ The axis scales are clearly visible. Include numbers along axes only for bottom left plot of group (a 'group' is an analysis of identical markers).

☒ All plots are contour plots with outliers or pseudocolor plots.

☒ A numerical value for number of cells or percentage (with statistics) is provided.

### Methodology

**Sample preparation**: For fluorescence-activated cell sorting (FACS) and cytometry, cells were collected by centrifugation for 5 min at 500 g, re-

| | |
|---|---|
| Sample preparation | suspended in ice-cold Sorting Medium (1% Fetal Bovine Serum in PBS, 0.25mg/mL Fungizone (Thermo Fisher Scientific), 0.25µg/mL/10µg/mL Amphotericin B/Gentamicin (GIBCO)) and filtered using 5 mL polystyrene round-bottom tubes with cell-strainer caps (Falcon) before sorting and cytometry on FACSAria III Cell Sorter (BD Biosciences). For sorting, the cells were collected in 96-well plates with Conditional Medium (1:1 mixture of fresh complete medium and medium collected from proliferating cell cultures that is 0.45µm filtered, supplemented with 20% Fetal Bovine Serum, 0.25mg/mL Fungizone (Thermo Fisher Scientific), 0.25µg/mL/10µg/mL Amphotericin B/Gentamicin (GIBCO)). |
| Instrument | ACSAria III Cell Sorter (BD Biosciences) |
| Software | Data collection and gating was done in FlowJo software. Post-processing analysis was done in Floreada.io software. |
| Cell population abundance | The purity of samples and fluorescence thresholds were assessed separately in each FACS experiment by cross-comparison of samples to negative and positive controls - DMSO-treated non IFN-induced or IFN-reinducted samples, respectively. The abundance of cell population per sample across the experiments and samples was within 13 441 - 23 927 cells. |
| Gating strategy | Gating conditions were adjusted separately in each FACS experiment by estimation of cell doublet size and cross-comparison of samples to negative and positive controls - DMSO-treated non IFN-induced or IFN-reinducted samples, respectively. |

☒ Tick this box to confirm that a figure exemplifying the gating strategy is provided in the Supplementary Information.

# Magnetic resonance imaging

## Experimental design

| | |
|---|---|
| Design type | *Indicate task or resting state; event-related or block design.* |
| Design specifications | *Specify the number of blocks, trials or experimental units per session and/or subject, and specify the length of each trial or block (if trials are blocked) and interval between trials.* |
| Behavioral performance measures | *State number and/or type of variables recorded (e.g. correct button press, response time) and what statistics were used to establish that the subjects were performing the task as expected (e.g. mean, range, and/or standard deviation across subjects).* |

## Acquisition

| | |
|---|---|
| Imaging type(s) | *Specify: functional, structural, diffusion, perfusion.* |
| Field strength | *Specify in Tesla* |
| Sequence & imaging parameters | *Specify the pulse sequence type (gradient echo, spin echo, etc.), imaging type (EPI, spiral, etc.), field of view, matrix size, slice thickness, orientation and TE/TR/flip angle.* |
| Area of acquisition | *State whether a whole brain scan was used OR define the area of acquisition, describing how the region was determined.* |

Diffusion MRI ☐ Used ☐ Not used

## Preprocessing

| | |
|---|---|
| Preprocessing software | *Provide detail on software version and revision number and on specific parameters (model/functions, brain extraction, segmentation, smoothing kernel size, etc.).* |
| Normalization | *If data were normalized/standardized, describe the approach(es): specify linear or non-linear and define image types used for transformation OR indicate that data were not normalized and explain rationale for lack of normalization.* |
| Normalization template | *Describe the template used for normalization/transformation, specifying subject space or group standardized space (e.g. original Talairach, MNI305, ICBM152) OR indicate that the data were not normalized.* |
| Noise and artifact removal | *Describe your procedure(s) for artifact and structured noise removal, specifying motion parameters, tissue signals and physiological signals (heart rate, respiration).* |
| Volume censoring | *Define your software and/or method and criteria for volume censoring, and state the extent of such censoring.* |

## Statistical modeling & inference

| | |
|---|---|
| Model type and settings | *Specify type (mass univariate, multivariate, RSA, predictive, etc.) and describe essential details of the model at the first and second levels (e.g. fixed, random or mixed effects; drift or auto-correlation).* |
| Effect(s) tested | *Define precise effect in terms of the task or stimulus conditions instead of psychological concepts and indicate whether ANOVA or factorial designs were used.* |

Specify type of analysis: ☐ Whole brain ☐ ROI-based ☐ Both

Statistic type for inference

(See Eklund et al. 2016)

*Specify voxel-wise or cluster-wise and report all relevant parameters for cluster-wise methods.*

Correction

*Describe the type of correction and how it is obtained for multiple comparisons (e.g. FWE, FDR, permutation or Monte Carlo).*

## Models & analysis

| n/a | Involved in the study |
| --- | --- |
| ☐ | ☐ Functional and/or effective connectivity |
| ☐ | ☐ Graph analysis |
| ☐ | ☐ Multivariate modeling or predictive analysis |

Functional and/or effective connectivity

*Report the measures of dependence used and the model details (e.g. Pearson correlation, partial correlation, mutual information).*

Graph analysis

*Report the dependent variable and connectivity measure, specifying weighted graph or binarized graph, subject- or group-level, and the global and/or node summaries used (e.g. clustering coefficient, efficiency, etc.).*

Multivariate modeling and predictive analysis

*Specify independent variables, features extraction and dimension reduction, model, training and evaluation metrics.*
