# Supplementary figures and images for: Heritable maintenance of chromatin modifications confers transcriptional memory of interferon-γ signaling
Source: Nat Struct Mol Biol. 2025 Apr 4;32(7):1255–67. doi: 10.1038/s41594-025-01522-8 (PMC12263432; doi:10.1038/s41594-025-01522-8)

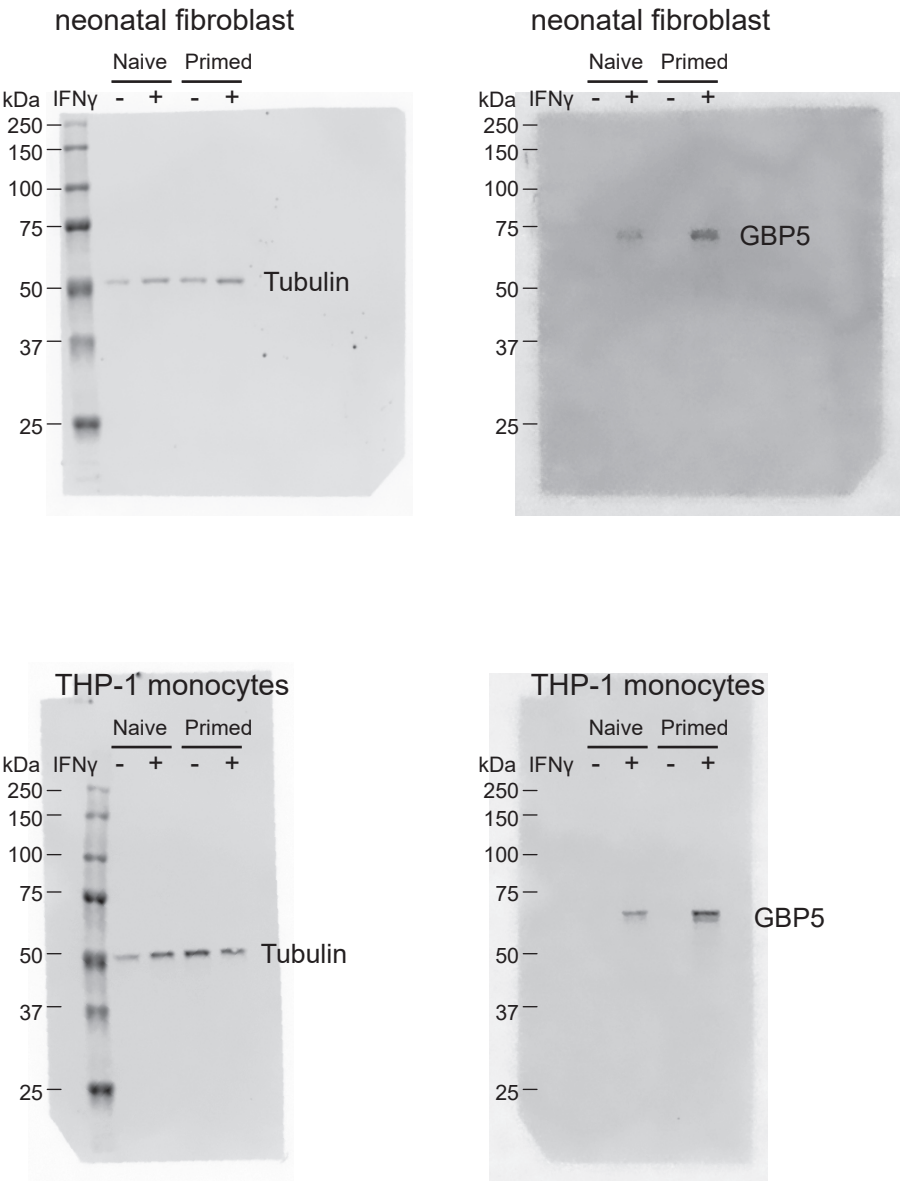

Supplement: Supplementary file 11 — Unprocessed western blots. [file 41594_2025_1522_MOESM11_ESM.pdf]

Source Data for Extended Data Fig. 8a

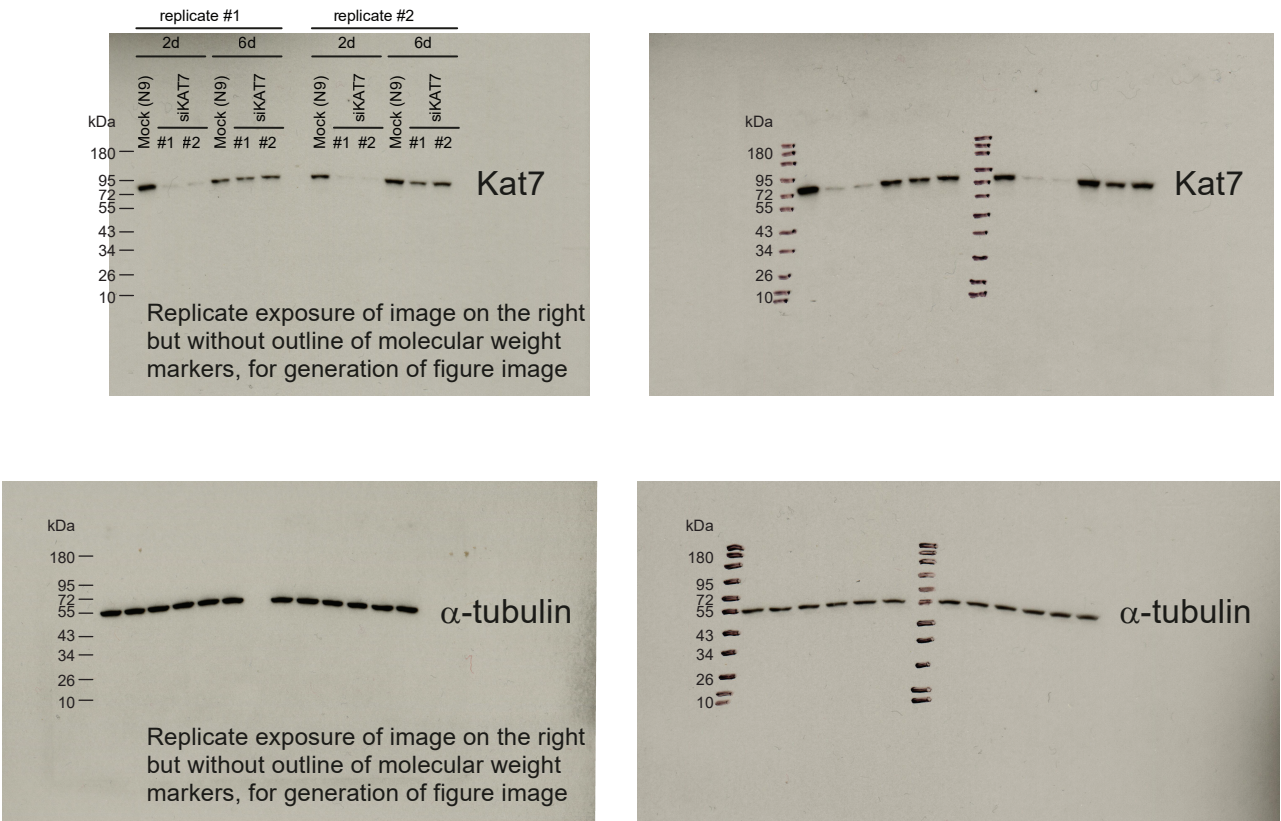

Source Data for Extended Data Fig. 8c

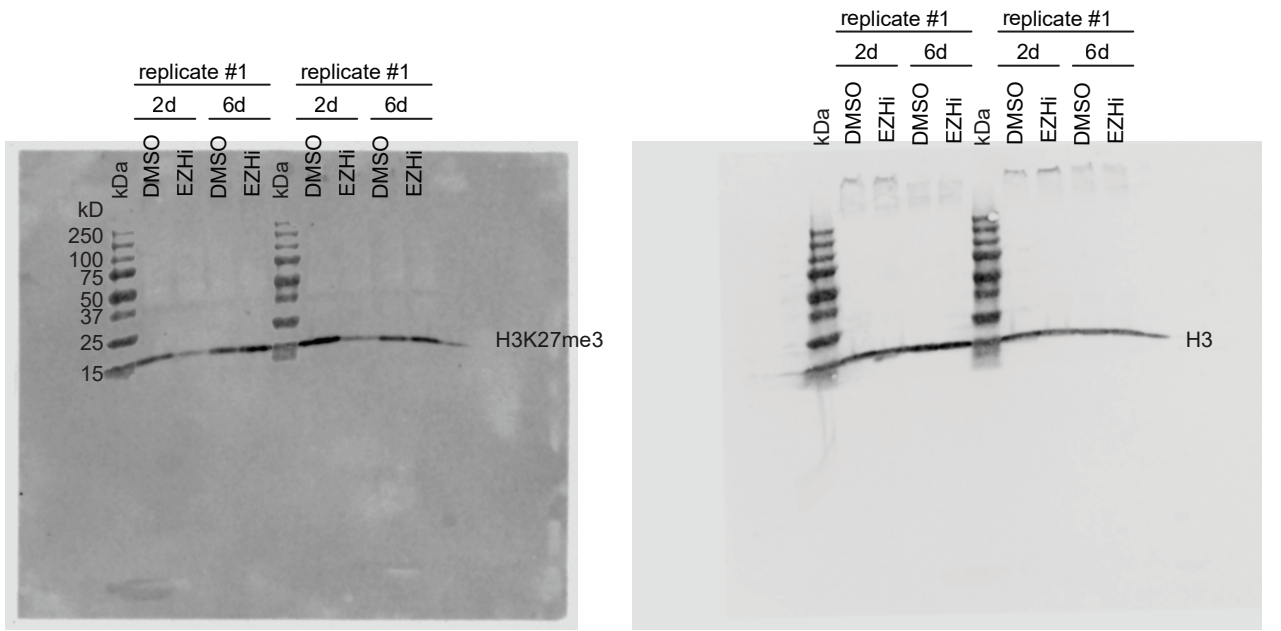

Supplement: Supplementary file 16 — Unprocessed western blots. [file 41594_2025_1522_MOESM16_ESM.pdf]
